# A deep learning system for predicting time to progression of diabetic retinopathy

**DOI:** 10.1038/s41591-023-02702-z

**Published:** 2024-01-04

**Authors:** Ling Dai, Bin Sheng, Tingli Chen, Qiang Wu, Ruhan Liu, Chun Cai, Liang Wu, Dawei Yang, Haslina Hamzah, Yuexing Liu, Xiangning Wang, Zhouyu Guan, Shujie Yu, Tingyao Li, Ziqi Tang, Anran Ran, Haoxuan Che, Hao Chen, Yingfeng Zheng, Jia Shu, Shan Huang, Chan Wu, Shiqun Lin, Dan Liu, Jiajia Li, Zheyuan Wang, Ziyao Meng, Jie Shen, Xuhong Hou, Chenxin Deng, Lei Ruan, Feng Lu, Miaoli Chee, Ten Cheer Quek, Ramyaa Srinivasan, Rajiv Raman, Xiaodong Sun, Ya Xing Wang, Jiarui Wu, Hai Jin, Rongping Dai, Dinggang Shen, Xiaokang Yang, Minyi Guo, Cuntai Zhang, Carol Y. Cheung, Gavin Siew Wei Tan, Yih-Chung Tham, Ching-Yu Cheng, Huating Li, Tien Yin Wong, Weiping Jia

**Affiliations:** 1 Shanghai Belt and Road International Joint Laboratory for Intelligent Prevention and Treatment of Metabolic Disorders, Department of Computer Science and Engineering, School of Electronic, Information, and Electrical Engineering, Shanghai Jiao Tong University, Department of Endocrinology and Metabolism, Shanghai Sixth People’s Hospital Affiliated to Shanghai Jiao Tong University School of Medicine, Shanghai Diabetes Institute, Shanghai Clinical Center for Diabetes, Shanghai, China; 2 MOE Key Laboratory of AI, School of Electronic, Information, and Electrical Engineering, Shanghai Jiao Tong University, Shanghai, China; 3 Department of Ophthalmology, Huadong Sanatorium, Wuxi, China; 4 Department of Ophthalmology, Shanghai Sixth People’s Hospital Affiliated to Shanghai Jiao Tong University School of Medicine, Shanghai, China; 5 Department of Ophthalmology and Visual Sciences, The Chinese University of Hong Kong, Hong Kong, China; 6 Singapore Eye Research Institute, Singapore National Eye Centre, Singapore, Singapore; 7 Department of Computer Science and Engineering, The Hong Kong University of Science and Technology, Hong Kong, China; 8 Department of Chemical and Biological Engineering, The Hong Kong University of Science and Technology, Hong Kong, China; 9 State Key Laboratory of Ophthalmology, Zhongshan Ophthalmic Center, Sun Yat-sen University, Guangdong Provincial Key Laboratory of Ophthalmology and Visual Science, Guangzhou, China; 10 Department of Ophthalmology, Peking Union Medical College Hospital, Peking Union Medical College, Chinese Academy of Medical Sciences, Beijing, China; 11 Medical Records and Statistics Office, Shanghai Sixth People’s Hospital Affiliated to Shanghai Jiao Tong University School of Medicine, Shanghai, China; 12 Department of Geriatrics, Tongji Hospital, Tongji Medical College, Huazhong University of Science and Technology, Wuhan, China; 13 National Engineering Research Center for Big Data Technology and System, Services Computing Technology and System Lab, Cluster and Grid Computing Lab, School of Computer Science and Technology, Huazhong University of Science and Technology, Wuhan, China; 14 Shri Bhagwan Mahavir Vitreoretinal Services, Medical Research Foundation, Sankara Nethralaya, Chennai, India; 15 Department of Ophthalmology, Shanghai General Hospital, Shanghai Jiao Tong University School of Medicine, Shanghai, China; 16 Beijing Institute of Ophthalmology, Beijing Tongren Eye Center, Beijing Tongren Hospital, Capital Medical University, Beijing Ophthalmology and Visual Science Key Laboratory, Beijing, China; 17 Center for Excellence in Molecular Science, Chinese Academy of Sciences, Shanghai, China; 18 School of Biomedical Engineering, Shanghai Tech University, Shanghai, China; 19 Shanghai United Imaging Intelligence, Shanghai, China; 20 Shanghai Clinical Research and Trial Center, Shanghai, China; 21 Ophthalmology and Visual Sciences Academic Clinical Program, Duke-NUS Medical School, Singapore, Singapore; 22 Centre for Innovation and Precision Eye Health; and Department of Ophthalmology, Yong Loo Lin School of Medicine, National University of Singapore, Singapore, Singapore; 23 Tsinghua Medicine, Beijing Tsinghua Changgung Hospital, Tsinghua University, Beijing, China

**Keywords:** Diabetes complications, Predictive markers, Machine learning

## Abstract

Diabetic retinopathy (DR) is the leading cause of preventable blindness worldwide. The risk of DR progression is highly variable among different individuals, making it difficult to predict risk and personalize screening intervals. We developed and validated a deep learning system (DeepDR Plus) to predict time to DR progression within 5 years solely from fundus images. First, we used 717,308 fundus images from 179,327 participants with diabetes to pretrain the system. Subsequently, we trained and validated the system with a multiethnic dataset comprising 118,868 images from 29,868 participants with diabetes. For predicting time to DR progression, the system achieved concordance indexes of 0.754–0.846 and integrated Brier scores of 0.153–0.241 for all times up to 5 years. Furthermore, we validated the system in real-world cohorts of participants with diabetes. The integration with clinical workflow could potentially extend the mean screening interval from 12 months to 31.97 months, and the percentage of participants recommended to be screened at 1–5 years was 30.62%, 20.00%, 19.63%, 11.85% and 17.89%, respectively, while delayed detection of progression to vision-threatening DR was 0.18%. Altogether, the DeepDR Plus system could predict individualized risk and time to DR progression over 5 years, potentially allowing personalized screening intervals.

## Abstract (web summary)

A deep learning algorithm shows promising performance in predicting progression to diabetic retinopathy in patients, up to 5 years in advance, potentially providing support for medical treatment decisions and indications for personalized screening frequency in a real-world cohort.

## Main

DR is the most common microvascular complication of diabetes and the leading cause of preventable blindness in adults aged 20–74 years^1–3^. Notably, DR mainly develops and progresses asymptomatically in the early stages until loss of vision occurs in the later stages of disease^2^.

However, the risk of DR progression is highly variable among different individuals, influenced by many modifiable and non-modifiable risk factors^4,5^. Currently, it is not possible to identify which patients with diabetes would develop DR or progress faster or slower. Consequently, routine screening for DR at yearly intervals is widely recommended for all individuals with diabetes with no DR or mild DR by national and international organizations^6–9^. Additionally, many individuals with diabetes are referred for monitoring and follow-up in specialist eye clinics or hospitals before they progress to severe DR, sometimes within just 2 years into a screening program^10,11^. Although previous studies have shown that as a group, DR is generally a slowly progressing disease^12^, and it is feasible to approximate progression risk for subgroups of patients with similar risk factors and DR severity levels^13^, it has been challenging to extend screening intervals from 1 year to 2 years (or even 3 years)^14^ because of the difficulty in accurately predicting an individual’s risk of and time to development of DR. As a result, many physicians and national DR screening programs have been extremely hesitant to recommend such an approach, although it would be highly cost-effective^14^.

Thus, one of the main challenges in managing DR is the lack of an individualized risk model and accurate prediction of the time to the onset and progression of the disease. By developing such a model, we can better estimate the risk and time frame for developing DR, which would significantly enhance the efficiency of DR screening programs^15^. Furthermore, we can allocate more intensive DR management strategies to those at high risk, which could help prevent the progression of DR.

Artificial intelligence (AI) has been playing an increasingly important role in medicine^2,16^. Deep learning (DL), with convolutional neural networks, has been developed for the automated detection of DR from retinal photographs^17–20^. There are, however, very few studies with retinal image-based DL systems to prospectively predict the risk of DR^15,21^. Moreover, there are critical gaps in existing research. First, regarding risk prediction of DR onset and progression, previous DL models focused on risk stratification within only 2 years after the baseline visit^15,21^. This is insufficient for a chronic disease such as DR because most patients do not develop DR progression within 2 years^5^. Second, an automated prediction of an individual’s time to DR onset and progression has not been explored in previous studies. Third, studies are needed to evaluate the impact of retinal image-based DL systems on patient outcomes when integrated into clinical workflow. These gaps need to be addressed before retinal image-based DL systems can be incorporated into DR screening programs.

We have previously developed a DL system (DeepDR), that can detect early-to-late stages of DR^20^. In the present study, we developed, validated and externally tested a DL system (DeepDR Plus), to predict individualized patient trajectories for DR progression within 5 years. Firstly, 717,308 fundus images from 179,327 patients with diabetes were used to pretrain the DeepDR Plus system. Subsequently, we trained and validated our DeepDR Plus system using clinical metadata and retinal fundus images from diverse multiethnic multicountry datasets, which comprise more than 118,868 images collected from 29,868 participants. To further demonstrate the outcome of the integration with clinical and digital workflows, we conducted a real-world study within prospective cohorts with diabetes.

## Results

### Study design and participants

**Fig. 1:**
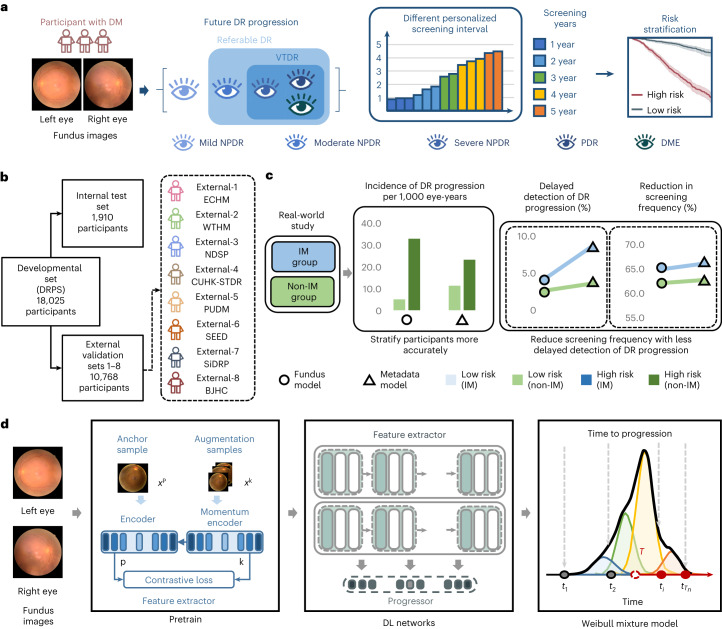
Design of the DeepDR Plus system. **a**, Schematic overview of the DeepDR Plus system. DeepDR Plus can predict the time to DR progression and perform risk stratification using retinal images of individuals with diabetes. **b**, Evaluation and application of the AI system. We trained our AI system on a developmental dataset and tested the generalizability in eight longitudinal independent cohorts. **c**, Schematic overview of the real-world study. **d**, Visual diagram of the DeepDR Plus system. DM, diabetes mellitus; DR, diabetic retinopathy; NPDR, non-proliferative diabetic retinopathy; PDR, proliferative diabetic retinopathy; DME, diabetic macular edema; DRPS, Diabetic Retinopathy Progression Study; ECHM, Eastern China Health Management; WTHM, Wuhan Tongji Health Management; NDSP, Nicheng Diabetes Screening Project; CUHK-STDR, Chinese University of Hong Kong-Sight-Threatening Diabetic Retinopathy; PUDM, Peking Union Diabetes Management; SEED, Singapore Epidemiology of Eye Diseases; SiDRP, Singapore National Diabetic Retinopathy Screening Program; BJHC, Beijing Healthcare Cohort Study; IM, integrated management; DL, deep learning. Source data

**Extended Data Fig. 1:**
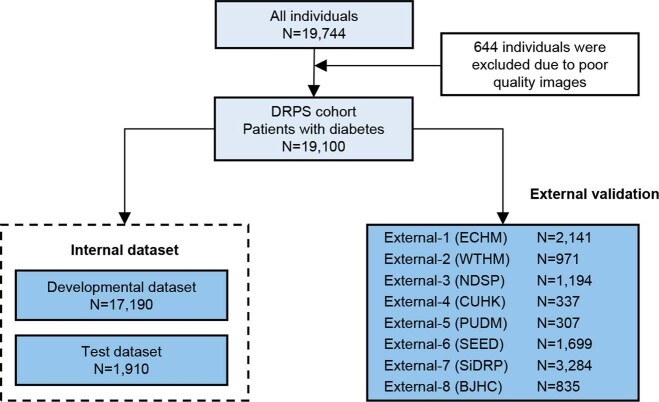
Datasets flowchart. DRPS, Diabetic Retinopathy Progression Study; ECHM, The Eastern China Health Management; WTHM, Wuhan Tongji Health Management; NDSP, Nicheng Diabetes Screening Project; CUHK-STDR, The Chinese University of Hong Kong-Sight-Threatening Diabetic Retinopathy; PUDM: Peking Union Diabetes Management; SEED, the Singapore Epidemiology of Eye Diseases study; SiDRP, the Singapore National Diabetic Retinopathy Screening Program; BJHC, Beijing Healthcare Cohort Study.

To learn the features associated with DR, the DeepDR Plus system was pretrained using 717,308 fundus images from 179,327 individuals with diabetes from the Shanghai Integrated Diabetes Prevention and Care System (Shanghai Integration Model)^20,22^ and the Shanghai Diabetes Prevention Program (SDPP). Subsequently, it was developed and validated in an internal dataset consisting of 76,400 fundus images from 19,100 individuals with diabetes collected from the Diabetic Retinopathy Progression Study (DRPS) cohort (Fig. 1). The DRPS cohort was divided into a developmental dataset and an internal test set at the patient level at a 9:1 ratio to predict the risk and time to DR progression at specific future time points. To validate the generalizability of the DeepDR Plus system, we used eight independent longitudinal cohorts for external validations (Methods and Extended Data Fig. 1). The baseline demographics information, anthropometric indices, biochemical measurements and retinal images of all the cohorts are summarized in Table 1. The relevant distribution of DR grades at baseline and at the end of follow-up in the developmental and validation datasets is shown (Extended Data Table 1), based on the International Clinical Diabetic Retinopathy Disease Severity Scale (ICDRDSS)^23^.

**Table 1:** Baseline characteristics of the participants in the internal dataset and external validation datasets

Cohorts	Pretrained dataset	Developmental dataset	Internal test set	ECHM External-1	WTHM External-2	NDSP External-3	CUHK-STDR External-4	PUDM External-5	SEED External-6	SiDRP External-7	BJHC External-8
Number of images	717,308	68,760	7,640	8,564	3,884	4,776	1,182	1,228	6,676	12,818	3,340
Number of participants	179,327	17,190	1,910	2,141	971	1,194	337	307	1,699	3,284	835
Race											
Chinese, *n* (%)	179,327 (100%)	17,190 (100%)	1,910 (100%)	2,141 (100%)	971 (100%)	1,194 (100%)	337 (100%)	307 (100%)	411 (24.19%)	2,494 (75.94%)	835 (100%)
Malay, *n* (%)	NA^a^	NA^a^	NA^a^	NA^a^	NA^a^	NA^a^	NA^a^	NA^a^	532 (31.31%)	518 (15.77%)	NA^a^
Indian, *n* (%)	NA^a^	NA^a^	NA^a^	NA^a^	NA^a^	NA^a^	NA^a^	NA^a^	756 (44.50%)	272 (8.28%)	NA^a^
Gender											
Female, *n* (%)	96,676 (53.91%)	2,692 (15.66%)	337 (17.62%)	826 (38.58%)	313 (32.23%)	739 (61.89%)	169 (50.15%)	131 (42.67%)	852 (50.15%)	1,661 (50.58%)	400 (47.90%)
Male, *n* (%)	82,651 (46.09%)	14,498 (84.34%)	1,573 (82.38%)	1,315 (61.42%)	658 (67.77%)	455 (38.11%)	168 (49.85%)	176 (57.33%)	847 (49.85%)	1,623 (49.42%)	435 (52.10%)
Age (years)	66.02 ± 8.47	58.23 ± 13.50	56.22 ± 12.14	57.00 ± 12.68	55.72 ± 11.60	62.04 ± 4.01	60.66 ± 12.45	52.09 ± 10.99	60.08 ± 9.40	58.14 ± 11.48	58.33 ± 12.44
Smoker, *n* (%)	19,249 (10.73%)	4,255 (24.75%)	470 (24.60%)	277 (12.94%)	215 (22.14%)	207 (17.34%)	83 (24.63%)	122 (39.74%)	484 (28.49%)	63 (1.9%)	NA^a^
Body mass index (kg/m^2^)	25.05 ± 3.11	25.96 ± 3.40	25.88 ± 3.41	26.23 ± 3.33	25.83 ± 3.54	25.93 ± 3.37	26.19 ± 4.84	25.38 ± 3.15	27.01 ± 4.67	27.96 ± 5.05	25.99 ± 3.62
Systolic blood pressure (mm Hg)	141.78 ± 18.80	134.29 ± 17.43	134.06 ± 17.19	129.77 ± 15.72	133.32 ± 16.50	139.04 ± 16.77	139.42 ± 20.62	123.75 ± 15.41	142.84 ± 20.46	133.09 ± 15.84	127.29 ± 16.398
HbA1c (%)	7.22 ± 1.24	6.95 ± 1.72	6.89 ± 1.67	7.05 ± 1.26	6.88 ± 1.59	6.76 ± 1.99	7.51 ± 1.34	7.40 ± 1.42	7.63 ± 1.61	6.72 ± 1.15	NA^a^
Fasting plasma glucose (mmol l^−1^)	7.66 ± 1.96	7.85 ± 2.18	7.83 ± 2.12	8.30 ± 2.11	7.59 ± 2.26	7.76 ± 1.97	7.76 ± 1.98	8.51 ± 2.67	NA^a^	7.51 ± 2.36	7.69 ± 2.22
Random blood glucose (mmol l^−1^)	NA^a^	NA^a^	NA^a^	NA^a^	NA^a^	NA^a^	NA^a^	NA^a^	9.85 ± 4.40	NA^a^	NA^a^
Triglycerides (mmol l^−1^)	1.64 ± 0.79	2.23 ± 2.07	2.18 ± 2.10	2.59 ± 2.42	2.28 ± 2.12	2.07 ± 1.86	1.47 ± 0.96	1.88 ± 2.19	2.06 ± 1.33	1.62 ± 1.02	1.90 ± 1.53
Low-density lipoprotein cholesterol (mmol l^−1^)	2.98 ± 0.88	3.09 ± 0.92	3.07 ± 0.91	3.08 ± 0.85	2.99 ± 0.92	3.25 ± 0.87	2.26 ± 0.72	2.67 ± 0.97	3.14 ± 0.96	2.68 ± 0.85	3.10 ± 0.93
High-density lipoprotein cholesterol (mmol l^−1^)	1.31 ± 0.32	1.25 ± 0.33	1.25 ± 0.33	1.21 ± 0.32	1.20 ± 0.32	1.29 ± 0.33	1.35 ± 0.47	1.22 ± 0.40	1.13 ± 0.32	1.29 ± 0.34	1.20 ± 0.35
Duration of diabetes mellitus (years)	NA^a^	5.03 ± 5.15	4.98 ± 5.22	5.50 ± 5.85	4.67 ± 4.13	5.90 ± 4.82	11.92 ± 9.42	7.41 ± 6.84	6.75 ± 7.95	1.41 ± 1.49	NA^a^
Eyes with DR progression, *n* (%)	NA^a^	2,819 (8.20%)	278 (7.28%)	321 (7.50%)	121 (6.23%)	48 (2.01%)	114 (16.91%)	25 (4.07%)	184 (5.41%)	171 (2.60%)	38 (2.28%)

Data are the mean ± s.d. or number of individuals (%). ^a^NA, not available.

### DeepDR Plus predicts time to DR progression

The DRPS cohort was used to develop the DL system for DR progression. In internal validation, for the prediction of DR progression among patients with diabetes, the metadata model achieved a concordance index (C-index) of 0.696 (95% confidence interval (CI), 0.668–0.725); the fundus model achieved a C-index of 0.823 (95% CI, 0.796–0.850), which was superior to the metadata model; and the combined model achieved a C-index of 0.833 (95% CI, 0.807–0.857). The aforementioned results indicated that the performance of the combined model was similar to that of the fundus model, demonstrating the accurate prediction performance of the fundus model (Extended Data Table 2). In the eight independent external datasets, the models achieved similar performances in predicting DR progression. The fundus model achieved C-indexes of 0.786–0.802, which were comparable with the combined model. The fundus model using low-resolution images of 128 × 128 pixels still yielded superior performance than the metadata model, suggesting that the resolution requirements for this technique can be easily met (Supplementary Fig. 1).

**Fig. 2:**
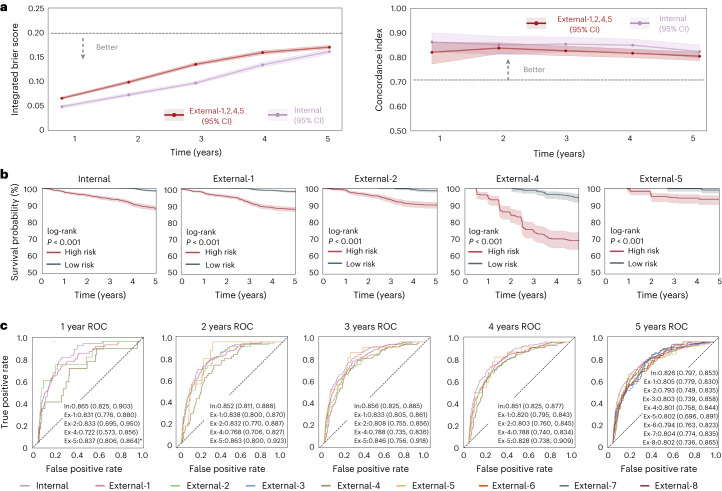
Internal and external validation of the fundus model in the prediction of the progression of DR. **a**, IBS (left) showing overall fit (lower is better) and C-index (right) measuring model risk discrimination (higher is better) for various time points. Data of external validation include retinal fundus images from individuals in the ECHM, WTHM, CUHK-STDR and PUDM cohorts. **b**, Kaplan–Meier plots for the prediction of DR progression. The *x* axis indicates the time in years. The *y* axis is the survival probability, measuring the probability of no DR progressing in 5 years. One-sided log-rank test was used for the comparison between the low-risk and high-risk groups. The *P* values for the internal test set and the external validation datasets 1, 2, 4 and 5 are 1.554 × 10^−41^, 3.258 × 10^−46^, 4.867 × 10^−17^, 2.946 × 10^−19^ and 1.888 × 10^−^^4^, respectively. **c**, Prediction of DR progression using time-dependent ROC curves. The asterisk indicates that there is only one case of the progression from non-DR to DR in the first year. The shaded areas in **a** and **b** denote 95% CIs. Areas under the ROC curves are presented as mean values (lower bound of 95% CI, upper bound of 95% CI). Source data

Subsequently, we predicted specific time to DR progression based on fundus images at years 1–5. C-index and the integrated Brier score (IBS) were used to evaluate the performance of the fundus model in the internal and external datasets. As illustrated in Fig. 2a, the fundus model achieved C-indexes of 0.823–0.862 and IBS ranged from 0.049 to 0.161 for years 1–5. The performance of the fundus model was carried over well to the external datasets 1, 2, 4 and 5, resulting in C-indexes of 0.804–0.837 and IBSs of 0.066–0.170, indicating the high concordance and strong calibration of the DeepDR Plus system.

**Extended Data Fig. 2:**
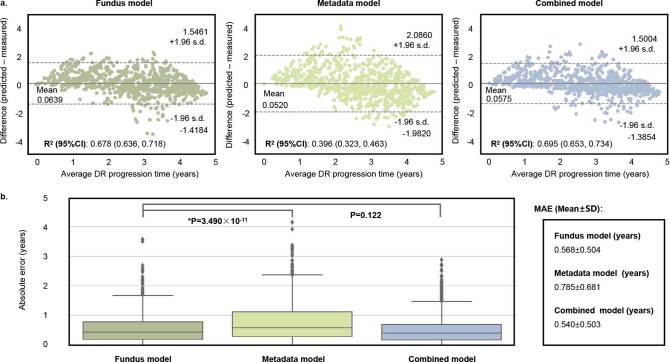
Model performance in predicting time to progression of eyes with DR progression in the internal test set and external validation dataset–1,2,4, and 5. **a**, Bland–Altman plots for the agreement between the predicted and actual time to DR progression. The *x* axis represents the mean of predicted and actual time to DR progression (average DR progression time), and the *y* axis represents the difference between the two measurements. **b**, Box plots show the distribution of samples for the absolute error for three models (the fundus model, the metadata model and the combined model) (n = 859). The horizontal line indicates the median and the whiskers indicate the lowest and highest points within the interquartile ranges of the lower or upper quartile, respectively. Mann-Whitney U test was used for the comparison among the models. R^2^, coefficient of determination; MAE, mean absolute error. Source data

To assess the prediction ability of the fundus model, we stratified eyes from individuals with diabetes into two groups (low or high risk) for DR progression according to the predicted risk scores. The threshold for the low-risk and high-risk groups was based on the median of the risk scores predicted from the fundus models in the developmental dataset. As shown in Fig. 2b, the fundus model can accurately discriminate low-risk and high-risk groups in both internal and external datasets (log-rank test *P* < 0.001). Additionally, we used time-dependent receiver operating characteristic (ROC) curves at years 1–5 to assess the prognostic accuracy of the fundus model of DR progression. For years 1–5, the areas under the ROC curve (AUCs) for DR progression ranged between 0.826 and 0.865 in the internal dataset. In the external sets, the AUCs ranged between 0.722 and 0.863 (Fig. 2c). Particularly, we showed the model performance in predicting time to progression of eyes with DR progression in the internal test set and external validation datasets 1, 2, 4 and 5 (Extended Data Fig. 2). The level of agreement between the predicted time to DR progression and the actual time to DR progression was depicted using a Bland–Altman plot. The fundus model demonstrated good performance in DR progression prediction, achieving a coefficient of determination (*R*^2^) of 0.678 (Extended Data Fig. 2a). The predictive performance of the metadata model (*R*^2^ = 0.396) was markedly lower compared with the fundus model. As shown in Extended Data Fig. 2b, the fundus model also resulted in a significantly lower mean absolute error compared with the metadata model (*P* < 0.001) and demonstrated no significant difference compared with the combined model (*P* = 0.122).

Furthermore, we conducted a subgroup analysis to evaluate the predictive performance of the fundus model considering the glycemic control (Supplementary Table 1). No significant difference was observed in the model performance for predicting DR progression among patients with different glycemic control statuses, regardless of the addition of follow-up hemoglobin A1c (HbA1c) levels.

### DeepDR Plus predicts time to progression in three subgroups

Because determining when patients should seek out an ophthalmologist and assessing the extent of DR are key concerns for both clinicians and patients, we conducted three subgroup analyses to provide additional evidence of the predictive capabilities of the DeepDR Plus system. The three subgroups included diabetes with no retinopathy to DR (subgroup 1), non-referable DR to referable DR (subgroup 2), non-vision-threatening DR to vision-threatening DR (subgroup 3). Referable DR was defined as moderate non-proliferative diabetic retinopathy (NPDR) or worse, and/or diabetic macular edema (DME). Additionally, we defined VTDR as severe NPDR, proliferative diabetic retinopathy (PDR) and/or DME.

**Extended Data Fig. 3:**
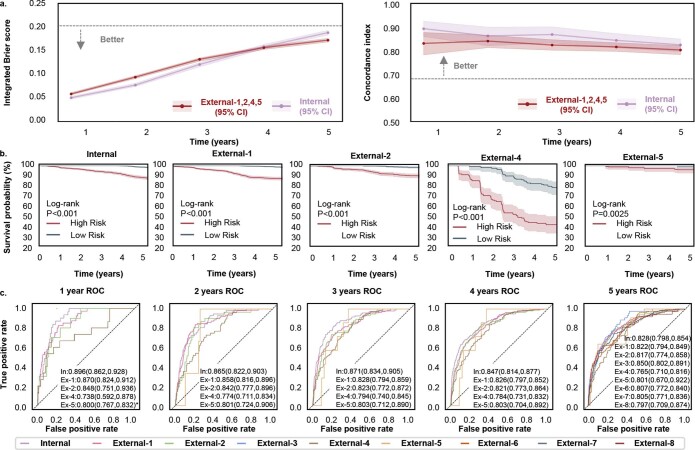
Internal and external validation of the DeepDR Plus system in the prediction of the progression from non-DR to DR. **a**, Integrated Brier score (left) showing overall fit—lower is better and C-index (right) measuring model risk discrimination—higher is better—for various time points. **b**, Kaplan–Meier plots for the prediction of the progression from non-DR to DR. One-sided log-rank test was used for the comparison between the low- and high-risk groups. The P values on internal test set and external validation dataset–1,2,4, and 5 are 6.638×10^−38^, 2.181×10^−46^, 5.453×10^−16^, 1.167×10^−12^ and 2.508×10^−3^, respectively. **c**, Prediction of the progression from non-DR to DR using time-dependent ROC curves. *The 1-year ROC of External-5 where only one case of the progression from non-DR to DR occurred that year. Shaded areas in **a** and **b** are 95% CIs. Areas under ROC curves are presented as mean values (lower bound of 95% CI, upper bound of 95% CI). Source data

**Extended Data Fig. 4:**
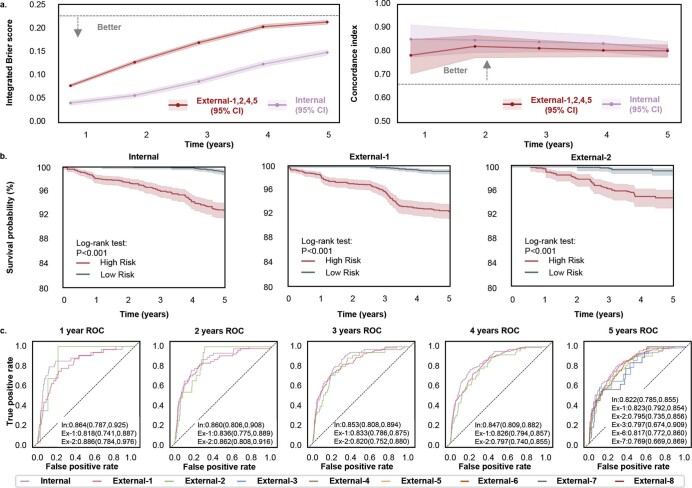
Internal and external validation of the DeepDR Plus system in the prediction of the progression from non-referable DR to referable DR. **a**, Integrated Brier score (left) showing overall fit—lower is better and C-index (right) measuring model risk discrimination—higher is better—for various time points. **b**, Kaplan–Meier plots for the prediction of the progression from non-referable DR to referable DR. One-sided log-rank test was used for the comparison between the low- and high-risk groups. The P values on internal test set and external validation dataset–1 and 2 are 6.995×10^-24^, 6.236×10^-28^ and 3.500×10^-9^, respectively. **c**, Prediction of the progression from non-referable DR to referable DR using time-dependent ROC curves. Shaded areas in **a** and **b** are 95% CIs. Areas under ROC curves are presented as mean values (lower bound of 95% CI, upper bound of 95% CI). Source data

**Extended Data Fig. 5:**
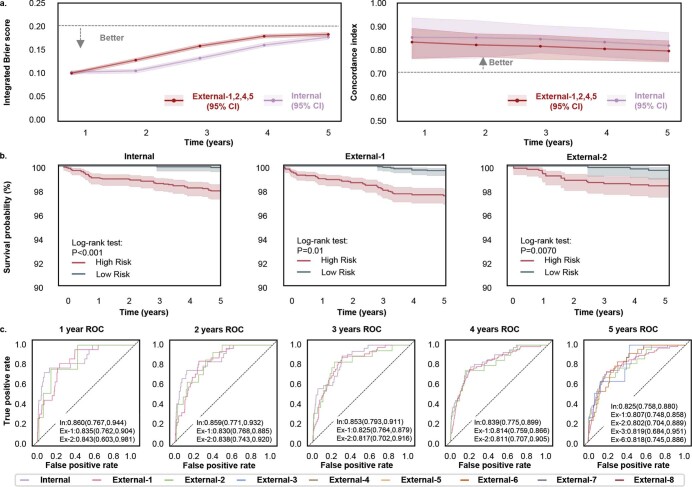
Internal and external validation of the DeepDR Plus system in the prediction of the progression from non-vision-threatening DR to vision-threatening DR. **a**, Integrated Brier score (left) showing overall fit—lower is better and C-index (right) measuring model risk discrimination—higher is better—for various time points. **b**, Kaplan–Meier plots for the prediction of the progression from non-vision-threatening DR to vision-threatening DR. One-sided log-rank test was used for the comparison between the low- and high-risk groups. The P values on internal test set and external validation dataset–1 and 2 are 8.528×10^-8^, 6.647×10^-8^ and 7.018×10^-3^, respectively. **c**, Prediction of the progression from non-vision-threatening DR to vision-threatening DR using time-dependent ROC curves. Shaded areas in **a** and **b** are 95% CIs. Areas under ROC curves are presented as mean values (lower bound of 95% CI, upper bound of 95% CI). Source data

In these three subgroups, we developed and tested the DeepDR Plus system using baseline retinal images to predict different types of DR grade deteriorations over 5 years. The performance of the three prediction models for each subgroup was compared using C-index and IBS. The metadata model achieved C-indexes of 0.700–0.711 and IBSs of 0.261–0.328 in predicting progression to any grade of DR, referable DR and VTDR in the internal dataset. Compared with the metadata model, C-indexes of the fundus model improved to 0.826 (95% CI, 0.797–0.851) for DR, 0.820 (95% CI, 0.785–0.853) for referable DR, and 0.824 (95% CI, 0.758–0.880) for VTDR, while IBS decreased to 0.153–0.189 for three subgroups. When the fundus images were combined with clinical metadata, the combined model gave C-indexes of 0.835–0.852 and IBS of 0.145–0.167 for subgroups 1–3 (Extended Data Table 2). Furthermore, we evaluated the prediction performance of the fundus model in the external datasets and achieved comparable results with the internal dataset (Extended Data Table 2). The results indicated that the fundus images alone could effectively predict the disease progression. Similarly to the task of predicting the time to any DR progression, we evaluated the prediction performance in three subgroups. As shown in Extended Data Figs. 3–5, the fundus model achieved C-indexes of 0.820–0.895 and IBSs of 0.045–0.189 in the internal dataset for years 1–5. Moreover, the external validation datasets 1, 2, 4 and 5 achieved C-indexes of 0.794–0.842 and IBSs of 0.058–0.218.

The risk stratification results of the DeepDR Plus system in predicting the progression of three subgroups are shown in Extended Data Figs. 3–5. We stratified baseline eyes from individuals with diabetes into two groups (low or high risk) for disease progression based on the predicted risk scores of the fundus model. Significant separations of the survival curves of each group were achieved in both internal and external datasets (*P* < 0.001) in three subgroups. Additionally, we used time-dependent ROC curves at years 1–5 to assess the prognostic accuracy of the fundus model for the above three situations. For years 1–5, the AUC values were 0.822–0.896 for predicting the onset of DR, referable DR and VTDR in the internal dataset. For external validation, the fundus model achieved comparable performance with AUC values ranging from 0.738 to 0.886.

### Applying the DeepDR Plus system improves clinical outcomes

**Extended Data Fig. 6:**
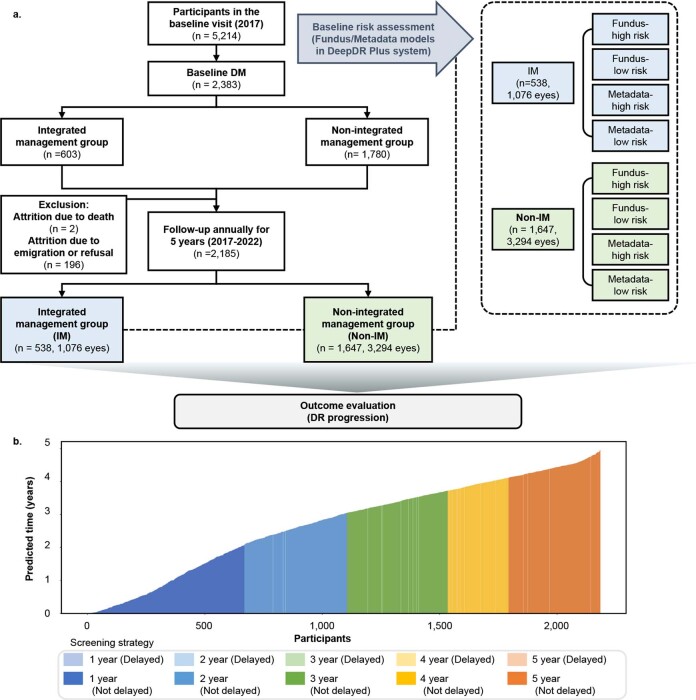
The real-world study to assess the clinical outcome by integration with DeepDR Plus system. **a**, Flowchart of the study and actual DR progression rate among high-risk and low-risk evaluated by the Fundus and Metadata models in IM group and Non-IM group. **b**, Waterfall plot of predicted time to DR progression of participants in the real-world study by fundus model (DeepDR Plus). The waterfall plot displays the predicted time to DR progression of all participants in the real-world study by the fundus model. The individualized screening interval was set at an annual time point from baseline, which was just the year after the predicted patient-specific time to DR progression by the fundus model. Source data

To evaluate the effectiveness of the DeepDR Plus system with the integration of clinical workflows, we conducted a real-world study within a community-based prospective cohort study of Chinese adults (Methods). A total of 2,185 participants were included in the analysis, with 538 participants in the integrated management (IM) group (integrated hospital–community diabetes management program) and 1,647 participants in the non-IM group. Participants in the IM group were provided regular clinical and metabolic measurements, advised by specialists in comprehensive hospitals and received lifestyle guidance and peer support at community health service centers^24^. Participant enrollment is outlined in Extended Data Fig. 6a and specific characteristics of all participants at baseline and at the end of follow-up are listed in Supplementary Table 2. The baseline retinal images and metadata of all participants were assessed by two models (the fundus model and metadata model in DeepDR Plus system) to evaluate their risk of DR progression. Each model generated the predicted time as a risk score, which was compared to a model-specific threshold obtained in the developmental dataset. Consequently, both models divided the IM group and non-IM group into low-risk and high-risk groups.

We calculated the adjusted relative reduction (ARR)^25,26^ of DR progression rate between the fundus model and metadata model in the DeepDR Plus system (Table 2). After adjustment for patient demographics, medical history, anthropometric indices and biochemical measurements, in the IM group, the difference in the DR progression rate between the fundus model and metadata model was not statistically significant in both low-risk group (ARR −33.05%; 95% CI, −67.79–35.76%) and high-risk group (ARR 14.54%; 95% CI, −28.26–74.63%). However, patients from the fundus high-risk group of the non-IM group had a higher DR progression rate compared with metadata high-risk group (33.13 versus 23.37 per 1,000 eye-years). Participants identified by the fundus high-risk group were prone to develop DR progression when they did not receive integrated hospital–community management (ARR 61.36%; 95% CI, 25.36–109.91%). Interestingly, in patients of the non-IM group, the fundus low-risk group had a significantly lower rate of DR progression compared with the metadata low-risk group (ARR −91.63%; 95% CI, −93.91% to −89.06%). Under comprehensive interventions (that is, intensive intervention for the high-risk group and non-intensive intervention for the low-risk group), compared with the metadata model, the fundus model can relatively prevent 46.80% DR progression incidence (ARR 46.80%; 95% CI, 12.37–94.93%).

**Table 2:** Associations between risk identification model and participant outcomes

		Eyes with DR progression incidence per 1,000 eye-years (number of cases/number of eyes)		ARR^a^ (95% CI)
Integrated hospital–community diabetes management program	IM group (*n* = 1,076)	DeepDR Plus-low risk (AI-low)	Metadata-low risk (meta-low)	−33.05 (−67.79, 35.76)
		5.11 (16/626)	7.63 (24/629)	
		DeepDR Plus-high risk (AI-high)	Metadata-high risk (meta-high)	14.54 (−28.26, 74.63)
		26.67 (60/450)	23.27 (52/447)	
	Non-IM (*n* = 3,294)	DeepDR Plus-low risk (AI-low)	Metadata-low risk (meta-low)	−91.63 (−93.91, −89.06)
		5.01 (50/1,996)	11.34 (113/1,993)	
		DeepDR Plus-high risk (AI-high)	Metadata-high risk (meta-high)	61.36 (25.36, 109.91)
		33.13 (215/1,298)	23.37 (152/1,301)	
	Comprehensive interventions: [(AI-high + AI-low) − (meta-high + meta-low)] in IM group − [(AI-high + AI-low) − (meta-high + meta-low)] in non-IM group			46.80 (12.37, 94.93)
Sankara Nethralaya-Diabetic Retinopathy Epidemiology and Molecular Genetics Study^b^	IM group (*n* = 146)	DeepDR Plus-low risk (AI-low)	Metadata-low risk (meta-low)	−9.39 (−79.77, 287.41)
		4.08 (2/98)	4.49 (2/89)	
		DeepDR Plus-high risk (AI-high)	Metadata-high risk (meta-high)	20.48 (−70.93, 400.0)
		25.0 (6/48)	21.05 (6/57)	
	Non-IM group (*n* = 1,798)	DeepDR Plus-low risk (AI-low)	Metadata-low risk (meta-low)	−97.32 (−98.28, −96.32)
		5.24 (28/1,068)	13.0 (70/1,077)	
		DeepDR Plus-high risk (AI-high)	Metadata-high risk (meta-high)	43.13 (9.1, 87.18)
		44.11 (161/730)	33.01 (119/721)	
	Comprehensive interventions: [(AI-high + AI-low) − (meta-high + meta-low)] in IM group − [(AI-high + AI-low) − (meta-high + meta-low)] in non-IM group			88.74 (10.83, 330.25)

^a^ARR is reported as ‘median (95% CI)’ by bootstrapping. ^b^Only eyes with gradable fundus images in both baseline and follow-up visits in the Sankara Nethralaya-Diabetic Retinopathy Epidemiology and Molecular Genetics Study were included.

To further evaluate the outcome of the integration with clinical workflows, we additionally conducted a real-world study within an Indian prospective cohort (SN-DREAMS)^27^, among 992 patients with diabetes who underwent 4 years of follow-up (Supplementary Table 3). Compared to the metadata model, the fundus model could relatively prevent 88.74% DR progression incidence under comprehensive interventions (Table 2).

Furthermore, we evaluated the performance of the personalized screening regime recommended by the metadata model or fundus model, compared with fixed annual screening. Table 3 shows the average screening interval, reduction in screening frequency and rate of delayed detection of DR progression in both IM and non-IM groups. For all participants, the mean screening interval could be extended from 12 months to 31.97 months if all participants in both IM and non-IM groups followed the recommended personalized screening interval given by the fundus model. Compared with the metadata model, the fundus model can achieve a similar reduction in screening frequency (62.46% versus 5.72%) while maintaining obviously less delayed detection of any DR progression (1.05% versus 4.99%). Additionally, there was a lower rate of delayed detection of any DR progression in patients of the IM group compared with the non-IM group (0.37% versus 1.28%) using the screening interval recommended by the fundus model, which suggested that the DeepDR Plus system could guarantee a low possibility of delayed detection of DR progression regardless of future interventions. Extended Data Fig. 6b shows the waterfall plot of predicted time to DR progression of participants in the real-world study by the fundus model. If all participants in both IM and non-IM groups followed the recommended personalized screening interval given by the fundus model, the percentage of participants who were recommended to screen DR at 1–5 years was 30.62%, 20.00%, 19.63%, 11.85% and 17.89%, respectively, while delayed detection of progression to VTDR was only 0.18%. To sum up, compared with the metadata model, the fundus model could stratify participants more accurately to enable personalized interventions and reduce DR screening frequencies with less delayed detection of DR progression.

**Table 3:** Performance of personalized screening regime recommended by the metadata model or fundus model in DeepDR Plus, compared with fixed annual screening

Group	Model	Average screening interval (months)	Reduction in screening frequency (%)^a^	Delayed detection of any DR progression (%)^b^	Delayed detection of progression to VTDR (%)
IM	Metadata	34.06	64.77	1.86	0.93
	Fundus	31.54	61.95	0.37	0.37
Non-IM	Metadata	35.32	66.02	6.01	0.97
	Fundus	32.11	62.63	1.28	0.12
IM and non-IM	Metadata	35.01	65.72	4.99	0.96
	Fundus	31.97	62.46	1.05	0.18

The screening interval was set at an annual time point from baseline, which was just the year after the predicted participant-specific time to DR progression by the metadata model or fundus model.

^a^The resulting reduction in the annual number of screenings of the population when applying the personalized screening regime recommended by the metadata model or fundus model in DeepDR Plus, compared with fixed annual screening.

^b^The rate of delayed detection of DR progression when applying the personalized screening regime recommended by metadata model or fundus model in DeepDR Plus, compared with fixed annual screening.

### Explainability analysis

The interpretability of the DeepDR Plus system can shed insight into its diagnostic mechanism and enable broad adoption. To better understand how the DeepDR Plus system could predict DR progressions, we took three steps to ensure the relevance and interpretability of the resulting features.

**Fig. 3:**
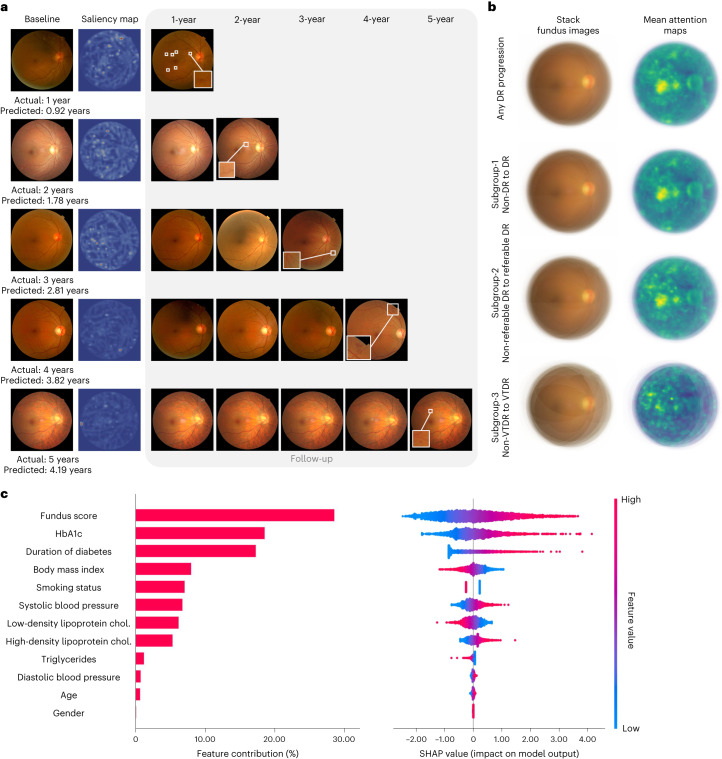
Explainability analysis of DeepDR Plus in predicting DR progression. **a**, Comparisons of color fundus photographs at baseline and follow-up using attention maps. **b**, Mean attention maps and corresponding stack fundus images for any DR progression and subgroups 1–3. **c**, Bar plot (left) of fundus score and clinical features and their contribution to the prediction model of DR progression. Features are in descending order by contribution (also known as importance) in the model. Details of associations are shown in a beeswarm plot (right) in which each point represents a participant. Color indicates the value of the feature, with red denoting higher and blue denoting lower. A negative SHAP value indicates negative feature attribution for the prediction of DR progression; a positive SHAP value indicates positive feature attribution for the prediction of DR progression. Source data

First, we conducted a saliency analysis using attention methods^28^ (Methods) to provide insights into the regions in the fundus images that could influence the predictions of the fundus model. Representative example attention maps at different times to DR progression (1 to 5 years, respectively) are shown in Fig. 3a. Attention maps of baseline fundus photographs were compared with annual follow-up fundus images. The results showed that our fundus model predicted DR progression by focusing on retinal vessels and the fovea. In addition, mean attention maps were generated for eyes with DR progression from the internal test set, reflecting that these observations were also generalized across many images (Fig. 3b).

It has been demonstrated that vessel density, fractal dimension and foveal avascular zone area could predict DR progression^29,30^. Previous studies also support that retinal vascular changes and related variables are associated with DR-related risk factors^31^. To further explore the patterns of retina associated with the future occurrence of DR, a range of retinal vascular variables were quantitatively measured by human graders who were masked to participant characteristics using our Singapore I vessel assessment software^32^. Cox regression analysis showed that higher venular fractal dimension in zone C was independently associated with any progression of DR, incident DR and incident referable DR after adjustment for age (*P* < 0.05) in the internal test set (Supplementary Tables 4 and 5). Meanwhile, central retinal vein equivalent in zone B and zone C significantly predicted the incidence of DR and referable DR (*P* < 0.05), and central retinal artery equivalent in zone B and zone C independently predicted incident referable DR (*P* < 0.05) after adjusting for age (Supplementary Table 5). Specifically, retinal vascular geometry resulted in a slight improvement in AUC values when added to the metadata model (Supplementary Table 6). These results showed that baseline retinal vascular geometry might be predictive patterns for the occurrence of DR, and the fundus model might pick up on signals beyond retinal vascular geometry to make these predictions.

Furthermore, we applied SHAP-based model interpretation to discover the predictive contribution of different clinical features for DR progression in the internal test set (Fig. 3c). The fundus score had the highest contribution to model performance.

## Discussion

Early screening and timely intervention are critical for the prevention and better clinical management of DR to achieve favorable outcomes. In this study, we developed a DL system called the DeepDR Plus system that utilized baseline retinal images to precisely predict individualized time to DR progression for all times up to 5 years. We also demonstrated the integration of this system into the clinical workflow could potentially extend the mean screening interval from the current 12 months to nearly 3 years, while delayed detection of progression to VTDR was only 0.18%. These results demonstrated that our DeepDR Plus system could potentially promote patient-specific risk assessment and further personalized care for DR management, based on just one single-time retinal check in the future.

The personalized interval for DR screening could improve the efficiency and put more attention on those who are at high risk for DR progression^33^, as in-person expert examinations are impractical and unsustainable given the pandemic size of the population with diabetes. In previous studies^15,21^, DL systems were created for predicting DR progression within 2 years, and were independent of available risk factors. Such a risk stratification tool might help to optimize screening intervals to reduce costs while improving vision-related outcomes. Considering the high variability in an individual’s risk of DR progression, our study provides a potential clinical tool to stratify low-risk and high-risk individuals with diabetes, which would support a personalized AI-driven approach to determine clinic follow-up intervals and more personalized management plans.

To further demonstrate the outcome of the integration of the DeepDR Plus system into clinical workflow, we first conducted a real-world study within a prospective cohort. Cutting-edge AI systems could not realize their full potential unless they are integrated into clinical and digital workflows^34^. In participants of the non-IM group, compared with the metadata model, participants with high risk identified by the DeepDR Plus system were prone to develop DR progression, suggesting that our DL models could predict patient-specific risk trajectories for DR progression more accurately than the metadata model. These participants could be preferentially selected for more intensive management or counseling^35,36^. Intriguingly, there was a significantly lower rate of DR progression in fundus low-risk group, which revealed that it might be relatively safe for these participants to achieve lenient control targets. Further, DeepDR Plus could potentially enable individualized DR screening intervals to balance early detection and reduce cost^14^. Compared with fixed annual screening for participants with no or mild NPDR, if all participants followed the recommended personalized screening interval given by the fundus model, the mean screening interval could be extended from 12 months to 31.97 months with 62.46% reduction in frequency, while the delayed detection of DR progression was minimal. Meanwhile, the DeepDR Plus system could also carry over well to Indians when integrated into clinical workflow, suggesting the generality of the system.

In our study, retinal vascular geometry and the fovea were important image patterns for future occurrence of DR based on the DeepDR Plus system, which was consistent with state-of-the-art studies and our previous studies^29–31,37,38^. Regions of DR-related capillary dropout have been related to local underlying photoreceptor loss, and the fovea may provide information on visual function and foveal perfusion^39,40^. It has also been demonstrated that changes in the caliber of the retinal vessel, especially widening of the venules, increase the risk of developing functional abnormalities in the eye and progression of DR^41^. Fractal dimension reflects underlying structural and/or functional alterations resulting from the effects of inflammation, neuronal abnormalities and other pathophysiological mechanisms^42–44^. In addition, increased vascular fractal dimension was associated with retinal neuropathy, which is an early event in the pathogenesis of DR^45–48^. The above studies might partially explain why retinal vascular geometry and the fovea are important image patterns for future occurrence of DR.

AI-based technology can assist DR screening, which is an unmet public health need^18–20,49^. Our study showed that an AI-driven personalized screening interval could be incorporated to improve efficiency, equity and accessibility of DR screening, particularly in low-resource settings^50^. Because highly effective recall systems can improve adherence to future clinical practice, integrating it into AI-based DR screening programs would further improve DR management. The use of AI in new classification of DR is promising^51^, and there are optimistic prospects for future guideline modifications pertaining to the use of AI in DR management.

Our study had several limitations. First, our DeepDR Plus system was trained in a Chinese population. Additional training on a wider variety of clinical and demographic datasets could improve predictive performance and its usefulness across multiple populations. Second, certain intrinsic biases, such as unidentified confounders (for example, myopia status), cannot be eradicated in the current retrospective study framework. Third, the performance of the fundus model may vary among patients with different treatment regimes, and this aspect still requires future testing for further ascertainment. Lastly, although the DeepDR Plus system was not actually applied to the clinic practice, our study could serve as proof of concept for developing large-scale personalized AI models for predicting DR progression and pave the way for future studies and randomized clinical trials to further evaluate the effectiveness of AI-driven DR screening and intervention. A foundation model for retinal images, named RETFound, was developed to provide a generalizable solution to improve model performance^52^. The integration of RETFound and DeepDR Plus system in the future may improve the predictive performance and may explore the application in early warning of other retinal diseases.

In summary, we developed DeepDR Plus, a system that could predict personalized risk and time to DR progression, solely based on baseline fundus images. The further real-world study showed that the integration of this system into the clinical workflow of patients could potentially extend the mean screening interval from the current 12 months to 31.97 months (nearly 3 years), with less delayed detection of DR progression. Thus, our DeepDR Plus system has great potential to integrate into clinical and digital workflows, in the hope of promoting individualized intervention strategies for DR management.

## Methods

### Ethical approval

This study was approved by the Ethics Committee of Shanghai Sixth People’s Hospital and conducted in accordance with the Declaration of Helsinki. Informed consent was obtained from all participants. The study was registered on the Chinese Clinical Trial Registry (ChiCTR2300069400).

### Data acquisition

To pretrain the DeepDR Plus system to make it learn features of DR, 717,308 fundus images of 179,327 individuals with diabetes from the Shanghai Integration Model^20,22^ and SDPP were used. The SDPP is a community-based, longitudinal cohort comprising 79,284 participants who underwent physical examinations between December 2015 and November 2022 in Huadong Sanatorium and Shanghai Sixth People’s Hospital. At baseline, data on demographic information, anthropometric indices, biochemical measurements and retinal images were recorded. After the baseline survey, 25,231 participants completed annual follow-up visits for at least 4 years.

To develop the DeepDR Plus system for predicting DR progression, fundus image data and clinical metadata were collected from the DRPS cohort. The DRPS cohort consists of two longitudinal cohorts. The dataset of the first cohort was extracted from the Hospital Information System of the Department of Endocrinology and Metabolism at Shanghai Sixth People’s Hospital and contained 15,587 patients with diabetes, who underwent annual health checks during a 5-year period. The second cohort enrolled 3,513 participants with diabetes at baseline from Huadong Sanatorium, and the cohort participants completed 5-year follow-up annually. Data on demographic information, anthropometric indices, biochemical measurements and retinal images were recorded at baseline and each visit. The diagnoses of DR grading and DME were based on the macular and optic disc-centered fundus images of each eye at baseline and follow-up visits.

We enrolled eight independent cohorts to serve as external validations. The ECHM (external dataset 1) is a community-based retrospective cohort study of participants who received comprehensive physical examination in Wuxi between 2006 and 2016. We enrolled 2,141 participants with diabetes who underwent annual examinations for 5 years in the ECHM. The WTHM cohort (external dataset 2) is a retrospective longitudinal cohort containing 971 participants with diabetes who received routine physical examinations at the physical examination center of the Geriatric Department of Tongji Hospital between 2010 and 2021. The NDSP cohort (external dataset 3) was a prospective observational study conducted in Nicheng, a large community in Shanghai, and the study was aimed at screening and following the progression of metabolic disorders and cardiovascular diseases among the older population of the entire area. The baseline survey was conducted in 2013, and 1,194 participants with diabetes completed the follow-up survey in 2018. The PUDM cohort (external dataset 5) was a clinic-based retrospective cohort, containing 307 participants with diabetes from the Peking Union Medical College Hospital, who received annual health checks between 2010 and 2016. The CUHK-STDR cohort (external dataset 4) was a prospective observational study involving 337 patients with diabetes^28^. Participants were recruited from CUHK Eye Centre in Hong Kong between July 2015 and November 2016 and had been consecutively followed up for at least 5 years. The SEED cohort (external dataset 6) is a multiethnic longitudinal population-based study including Singaporean adults of Malay, Indian and Chinese descent^18,53^. In total, 1,699 individuals with diabetes from the SEED cohort with 5-year follow-ups were enrolled for external validation. The SiDRP (external dataset 7) was a retrospective longitudinal cohort covering all 18 primary care clinics across Singapore from 2010 to 2015. It provided ‘real-time’ assessments of DR photographs by a centralized team of trained and accredited graders supported by a tele-ophthalmology information technology infrastructure^54^. A total of 3,284 individuals with diabetes from the cohort were enrolled for external validation. The BJHC (external dataset 8) is a community-based prospective study. In total, 835 patients with diabetes from the BJHC were included in this study. Baseline examinations were performed in the period between 2014 and 2016, and follow-up examinations were conducted between 2019 and 2020. In all external cohorts, two retinal photographs (macular and optic disc-centered) were captured for each eye at baseline and follow-up visits.

For the real-world study within a community-based prospective cohort study of Chinese adults, 5,214 participants were screened in March 2017, with participants without a self-reported history of diabetes undergoing a 75 g oral glucose tolerance test at baseline. Details of biochemical measurements and anthropometric data collection included body weight, waist circumference, blood pressure, lipid profile and related factors of cardiometabolic diseases. There were 2,383 participants with diabetes (non-DR or mild NPDR) enrolled in the final cohort according to World Health Organization 2019 criteria^55^, with 603 participants in the IM group and 1,780 participants in the non-IM group. Participants in the IM group were provided regular clinical and metabolic measurements, advised by specialists in comprehensive hospitals, and received lifestyle guidance and peer support at community health service centers^24^. Participants in this program were followed up annually for 5 years. In March 2022, 538 participants in the IM group and 1,647 participants in the non-IM group completed the follow-up visit.

### Diagnostic criteria

Diabetes is diagnosed by a self-reported history of diabetes, fasting plasma glucose ≥ 7.0 mmol l^−1^, 2-h plasma glucose ≥ 11.1 mmol l^−1^ and/or HbA1c ≥ 6.5%^56^. The diagnosis and classification of DR and DME were evaluated according to the ICDRDSS^23^. The progression of DR was defined as the first deterioration of DR grades or new onset of DME, based on ICDRDSS during the follow-up.

### Image quality control and grading procedure

For the DRPS, ECHM, WTHM, NDSP, PUDM, BJHC and Chinese real-world study datasets, the retinal fundus images were captured using a variety of standard fundus cameras, including Topcon TRC-NW6 (Topcon), Canon CR1–Mark II (Canon) and Optos camera (Optos). All fundus images were read by a centered reading group consisting of 12 certified ophthalmologists. Original retinal images were uploaded to an online platform^20^, and the images of each eye were assigned separately to 2 authorized ophthalmologists. They labeled the images using the online reading platform and gave the graded diagnosis of DR. The third ophthalmologist who served as the senior supervisor confirmed or corrected when the diagnostic results were contradictory. The final grading result was dependent on the consistency among these 3 ophthalmologists. The grading procedures for the CUHK-STDR^28^, SEED^18,53^, SiDRP^18^ and SN-DREAMS^27^ datasets are reported in previous publications. Ungradable images of all the datasets were excluded from the study.

### Model development and training

We developed the DeepDR Plus system to predict DR progression. The DeepDR Plus system contains three models for predicting DR progression: the metadata model, the fundus model and the combined model. The risk and time to DR progression are estimated based on baseline inputs. The fundus model has a feature extractor to extract features from fundus images (details in ‘Model pretraining’) and a predictor to generate fundus score by estimating the survival time given the input data (details in ‘Model evaluation’). The fundus model utilizes the ResNet-50 as the backbone to extract features from the fundus images, and a soft-attention layer is used to select the most informative features. The metadata model inputs the metadata to produce survival predictions. The output of the fundus model (fundus score) combined with metadata is used as the input of the combined model. Metadata includes age, gender, smoking status, duration of diabetes, baseline DR level, body mass index, glycated HbA1c, systolic blood pressure, diastolic blood pressure, triglycerides, low-density lipoprotein cholesterol and high-density lipoprotein cholesterol. We also developed and compared the metadata model, the fundus model and the combined model for predicting DR progression in three subgroups. The three subgroups included diabetes with non-DR to DR (subgroup 1), non-referable DR to referable DR (subgroup 2) and non-VTDR to VTDR (subgroup 3).

### DR progression model

**Extended Data Fig. 7:**
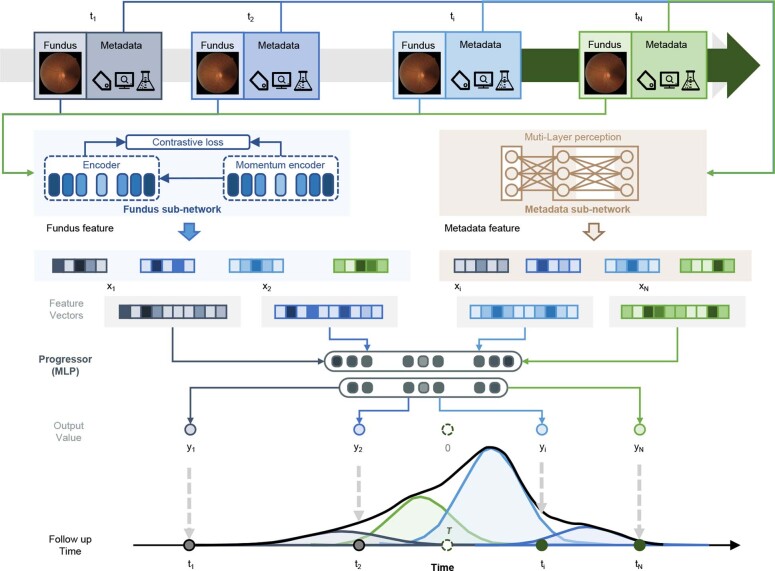
Illustration of the model structure of DeepDR Plus system. There are three models (fundus model, metadata model, and combined model) in the DeepDR Plus system, which can support different types of inputs. The fundus model has a fundus feature extractor and a predictor to generate a predicted time to progression of DR and fundus score. The fundus feature extractor is pretrained using Momentum Contrast (MoCo v2) to generate high-level feature vectors, while the predictor estimates the survival time in a fixed-size mixture of Weibull distributions based on the fundus feature vectors to generate the fundus score. The metadata and combined models share the same structure but differ in their inputs compared to the fundus model. The metadata model takes metadata as inputs, while the combined model takes both metadata and fundus score as inputs.

Considering that most of the longitudinal datasets in this study have a fixed follow-up exam period of about 1 year, the longitudinal dataset suffers from right censoring and interval censoring. The aim of the DR progression model is to estimate the survival function. To achieve this goal, we modeled the survival distribution of each individual object as a fixed-size mixture of Weibull distributions. The parameters from each Weibull distribution were randomly sampled and fixed. We used a deep learning network to estimate the weights for each distribution in the mixture model (a fixed-size mixture of Weibull distributions). During training, the parameters of the deep learning network were optimized by adjusting their values to maximize the likelihood, which represents the probability of the observed training data given by the DeepDR Plus system. The development of these algorithms is described in detail below (Extended Data Fig. 7).

#### Problem definition

We have a longitudinal dataset *S* containing multiple objects *s*, where each object ${s}_{i}=\langle {x}_{i},{t}_{i},{t}_{i}^{{\prime} },{e}_{i}\rangle$. Here, *x*_*i*_ represents the feature vector (including image features and/or metadata at baseline). *e*_*i*_ indicates whether the record is censored. In particular, ${e}_{i}=1$ for the uncensored records, and ${e}_{i}=0$ otherwise. *t*_*i*_ represents the time to the last exam before the event of interest. If the event is observed, then ${t}_{i}^{{\prime} }$ represents the time until that event occurred. However, if the event is not observed, then *t*_*i*_ is equal to ${t}_{i}^{{\prime} }$. We used a mixture of Weibull distributions to model the survival function $S(t)={\mathbb{P}}(T > t)={\int }_{t}^{{\infty }}f(u)du$ of each participant. As the Weibull distribution is only valid for positive reals, it is suitable for survival analysis. Besides, the Weibull distribution has an analytic solution for the cumulative distribution function, which enables the use of gradient-based optimization for maximum likelihood estimation in our research^57^.

Our goal is to estimate a set of parameters and weights for the mixture model given the input. The survival function of each patient is defined as follows:

$${\mathbb{P}}(T > t|x)=\mathop{\sum }\limits_{i=1}^{K}{\phi }_{i|x}\underset{t}{\overset{\infty }{\int }}{f}_{i}(u|{\alpha }_{i},{\beta }_{i})du$$

where ${f}_{i}(u|{\alpha }_{i},{\beta }_{i})=\frac{{\beta }_{i}}{{\alpha }_{i}}{(\frac{u}{{\alpha }_{i}})}^{{\beta }_{i}-1}\exp (-{(u/{\alpha }_{i})}^{{\beta }_{i}})$ is the probability distribution function of the Weibull distribution, and *α*_*i*_ and *β*_*i*_ are drawn from the Gaussian distribution $\log {\beta }_{i}\sim {\mathscr{N}}({\beta }_{0},1/\lambda )$, $\log {\alpha }_{i}\sim {\mathscr{N}}({\alpha }_{0},1/\lambda )$. *α*_0_, *β*_0_ and *λ* are prior parameters determined empirically. *ϕ* is a set of parameters for the mixture distribution containing multiple parameters ${\phi }_{i|x}$.

The cumulative distribution function of the Weibull distribution is given by

$${\mathbb{P}}(T\langle t|x)=\mathop{\sum }\limits_{i=1}^{K}{\phi }_{i|x}(1-\exp (-{(t/{\alpha }_{i})}^{{\beta }_{i}})).$$

The input feature matrix (including fundus images and clinical metadata) *I* is passed through the deep learning network $f(\cdot |{\boldsymbol{\Theta }})$ to determine all the parameters **Θ**.

We used a maximum likelihood estimation to estimate the parameters of the deep learning network. For uncensored data, the log-likelihood function can be written by

$$\begin{array}{c}\mathrm{ln}\,{\mathbb{P}}({{\mathscr{D}}}_{U}| {\boldsymbol{\Theta }})=\,\mathrm{ln}\left(\mathop{\prod }\limits_{i=1}^{|{{\mathscr{D}}}_{U}|}{\mathbb{P}}(T > {t}_{i}| X={x}_{i},{\boldsymbol{\Theta }}){\mathbb{P}}(T < {t}_{i}^{{\prime} }| X={x}_{i},{\boldsymbol{\Theta }})\right)\\ =\mathop{\sum }\limits_{i=1}^{|{{\mathscr{D}}}_{U}|}(\mathrm{ln}\,{\mathbb{P}}(T > {t}_{i}| X={x}_{i},{\boldsymbol{\Theta }})+\,\mathrm{ln}\,{\mathbb{P}}(T < {t}_{i}^{{\prime} }| X={x}_{i},{\boldsymbol{\Theta }})).\end{array}$$

Similarly, for censored data the log-likelihood function can be given by

$$\begin{array}{c}\begin{array}{cc}\mathrm{ln}\,{\mathbb{P}}({{\mathscr{D}}}_{C}| {\boldsymbol{\Theta }})=\,\mathrm{ln}\left(\mathop{\prod }\limits_{i=1}^{|{{\mathscr{D}}}_{C}|}{\mathbb{P}}(T > {t}_{i}| X={x}_{i},{\boldsymbol{\Theta }})\right) & \end{array}\\ =\mathop{\sum }\limits_{i=1}^{|{{\mathscr{D}}}_{C}|}(\mathrm{ln}\,{\mathbb{P}}(T > {t}_{i}| X={x}_{i},{\boldsymbol{\Theta }})).\end{array}$$

And we define the loss function as

$$L=-\mathop{\sum }\limits_{i=1}^{|{\mathscr{D}}|}\,\mathrm{ln}\,\mathop{\sum }\limits_{j=1}^{K}{\phi }_{j|x}(\exp (-{({t}_{i}/{\alpha }_{j})}^{{\beta }_{j}}))-\gamma \mathop{\sum }\limits_{i=1}^{|{\mathscr{D}}|}{e}_{i}\,\mathrm{ln}\,\mathop{\sum }\limits_{j=1}^{K}{\phi }_{j|x}(1-\exp (-{({t}_{i}^{{\prime} }/{\alpha }_{j})}^{{\beta }_{j}})),$$

where *γ* is a hyperparameter that balances the weight of censored data and uncensored data.

### Model pretraining

We used Momentum Contrast (MoCo, v2)^58,59^, which leverages self-supervised learning to produce a pretrained feature extractor. In this process, the feature extractor is trained on a large dataset of fundus images without the need for manual annotations. MoCo v2 uses a momentum-based contrastive learning framework, where the pretrain framework in the fundus model learns to create positive and negative pairs of image patches from the same image^59^. We laid out the experimental setup in our pretrain process. We predominantly followed the experimental settings of MoCo v2, but adopted different approaches in data augmentation and certain hyperparameter selections. We used the *k*-nearest neighbors monitor as a tool for self-supervised evaluation once per epoch. For data augmentation, our augmentation method included random image compression, random blur, brightness jitter, contrast jitter, random gamma transform, random Gaussian noise and random rotation. Two 512 × 512 crops were taken for each fundus image in each iteration. For hyperparameter selections, we chose ResNet-50 as the encoder and stochastic gradient descent (SGD) as the optimizer. The input image resolution was 512 × 512 pixels, the batch size was set to 256, and the MoCo v2 model was trained for 800 epochs. Grid search was used to obtain the optimal hyperparameters as a learning rate = 10^−^^3^, weight decay = 10^−^^4^, SGD momentum = 0.9 and temperature *τ* = 1.0. The encoder momentum coefficient was m = 0.996 and it was increased to 1 with a cosine schedule. We also conducted an ablation experiment to evaluate the predictive performance of the fundus model by pretraining with MoCo v2. The results showed that incorporating MoCo v2 into the training process could enhance the predictive performance of the fundus model (Supplementary Table 7).

### Fundus model

For tasks of predicting DR progression, we used the pretrained ResNet-50 as the feature extractor. The self-attention layer^60^ was used in our network to emphasize the important parts in fundus features. Specifically, we added a standard dot-product self-attention layer^61^ to calculate the weight of each pixel in the feature map produced by the block3 of ResNet-50. The attention layer outputs the feature map with the same size as the input feature map to ensure it can be inserted into ResNet-50 seamlessly. In the interpretation stage, the attention feature map was reshaped to 32 × 32 and subsequently resized to *H* × *W* for illustration. In addition, a three-layer multilayer perceptron (MLP) as a predictor was used to estimate the weights of the fixed-size mixture of Weibull distributions taking as input the features generated by the pretrained ResNet-50 model.

The fundus model was then trained on the training dataset using an SGD optimizer. In this study, we used grid search to find the optimal values and other hyperparameters. The fundus model was trained by back-propagation of errors in batches of 32 images resized to 512 × 512 pixels for 50 epochs with a learning rate of 10^−^^5^. Data augmentation strategies used here were the same as those used during MoCo v2-based pretraining (details in ‘Model pretraining’).

### Metadata model and combined model

We used a three-layer MLP in the metadata model and combined model. The metadata model takes the metadata as input and outputs the predicted time-to-event for the individual participant. For the combined model, the predicted time-to-event from the fundus model is added as an extra feature for the three-layer MLP. The metadata model, fundus model and combined model share the same loss function during the model development.

### Model evaluation

To estimate the time of the target event, we first calculated the survival function using the baseline input, then we took the predicted time at the maximum point of the density function as the predicted time to event. Using this predicted time-to-event as the risk score, we evaluated the performance of the model in predicting whether the given participant would have the target disease within 1, 2, 3, 4, or 5 years, using C-index and IBS. According to the scores of the baseline visit obtained from the fundus model, the participants were triaged into two groups: low and high risk according to the threshold defined by the upper and lower half of the predicted scores in groups of participants with different DR progression outcomes. Kaplan–Meier curves were constructed for the risk groups, and the significance of differences between group curves was computed using the log-rank test. Time-dependent ROC curves were used to quantify model performance on validation sets at the time of interest. ROC curves were constructed at a landmark time from predicted risk scores of relative participants using the DeepDR Plus system.

### Interpretation of AI predictions

A visualization tool is needed that would enable clinicians to understand important clinical visual features in fundus images. To this end, following Google’s approach^28^, we first produced individual attention maps as visual explanations by inserting a self-attention layer into the architecture of the fundus model (details in ‘Fundus model’). The most predictive features captured by the DeepDR Plus system were highlighted for each individual image. To generate mean attention maps, all individual fundus images and individual attention maps were aligned based on their optic disc positions. That is, all fundus images and attention maps were translated to share the same optic disc position. Subsequently, final mean attention maps were obtained by averaging the ‘registered’ individual attention weights across multiple images. What’s more, we used the SHAP Python package^62^ to illustrate the importance of clinical features as well as the fundus score (that is, predicted time-to-event by fundus model) involved in the combined model. SHAP stands for Shapley Additive exPlanations^63^. The SHAP values of each feature represented their contribution to the model prediction. A positive SHAP value indicates the positive feature attribution for the prediction of DR progression, whereas a negative SHAP value indicates the negative feature attribution for the prediction of DR progression. Feature importance was calculated by averaging the absolute SHAP values of each feature.

### Reporting summary

Further information on research design is available in the Nature Portfolio Reporting Summary linked to this article.

## Online content

Any methods, additional references, Nature Portfolio reporting summaries, source data, extended data, supplementary information, acknowledgements, peer review information; details of author contributions and competing interests; and statements of data and code availability are available at 10.1038/s41591-023-02702-z.

### Supplementary information

Supplementary Information Supplementary Fig. 1 and Supplementary Tables 1–7

Reporting Summary

### Source data

Source Data Figs. 1–3 Statistical source data for Figs.1c, 2a–c and 3c.

Source Data Extended Data Figs. 2–6 Statistical source data for Extended Data Figs. 2a,b, 3a–c, 4a–c, 5a–c and 6b.

## Figures and Tables

**Extended Data Table 1:**
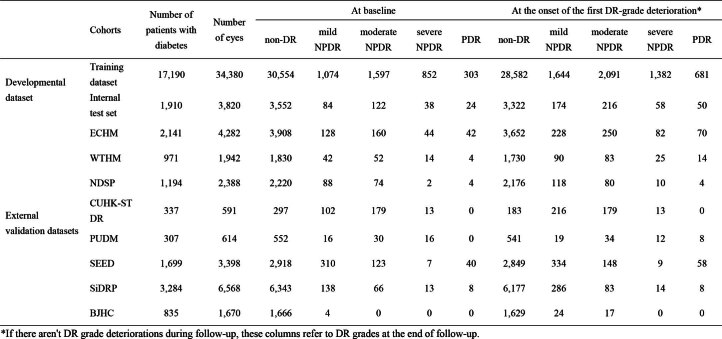
The distribution of diabetic retinopathy grades at baseline and the onset of the first DR grade deterioration in the developmental and validation datasets

**Extended Data Table 2:**
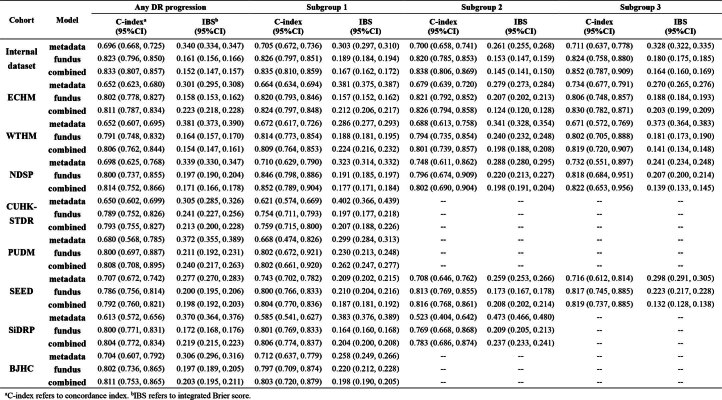
Performance of the prediction model of DR progression based on metadata model, fundus model, and combined model in internal and external validation datasets

## Data Availability

Individual-level patient data can be accessible with the consent of the data management committee from institutions and are not publicly available. Requests for the non-profit use of the fundus images and related clinical information should be sent to W.J. or T.Y.W. The data management committee will then review all the requests and grant (if successful). A formal data transfer agreement will be required upon approval. Generally, all these requests for access to the data will be responded to within 1 month. All data shared will be de-identified. For the reproduction of our algorithm code, we have also deposited a minimum dataset at Zenodo (https://zenodo.org/records/10076339), which is publicly available for scientific research and non-commercial use. Source data are provided with this paper.

## Code availability

The code used in the current study for developing the algorithm is provided at https://github.com/drpredict/DeepDR_Plus. Python version 3.9.0 was used for all statistical analvses in this study. The following third-party Python packages were used: Pytorch version 2.0.1 was used to build the DL models; Scikit-earn version 1.3.0 was used for calculating AUC. NumPy version 1.25.2 used for calculating C-index and Brier score. Lifelines version 0.27.7 was used for survival analysis.
