# Supplementary material for: A deep learning system for predicting time to progression of diabetic retinopathy
Source: Nat Med. 2024 Jan 4;30(2):584–94. doi: 10.1038/s41591-023-02702-z (PMC10878973; doi:10.1038/s41591-023-02702-z)
Supplement: Supplementary file 1 — Supplementary Fig. 1 and Supplementary Tables 1–7 [file 41591_2023_2702_MOESM1_ESM.pdf]

nature medicine

Article

https://doi.org/10.1038/s41591-023-02702-z

A deep learning system for predicting time to progression of diabetic retinopathy

In the format provided by the authors and unedited

A deep learning system for predicting time to progression of diabetic retinopathy

Supplementary Information

Contents

Supplementary Table 1. Performance of predicting DR progression in the fundus model with or without addition of HbA1c level at first follow-up visit by different glycemic control statuses in the internal test set……………………………………………………………………………………2

Supplementary Table 2. Clinical characteristics of participants in the Chinese real-world study at baseline and follow-up……………………………………………………………………………3

Supplementary Table 3. Clinical characteristics of participants in the Indian real-world study at baseline and follow-up……………………………………………………………………………5

Supplementary Table 4. Baseline retinal vascular measures between participants with and without any progression of DR, incidence of DR, referable DR, and VTDR…………………………………...7

Supplementary Table 5. Relationships between retinal vascular measures (per SD increase) and any progression of DR, incidence of DR, referable DR, and VTDR………………………………………..8

Supplementary Table 6. Performance of adding retinal vascular geometry to the metadata model for predictive tasks in the internal test set…………………………………………………….............9

Supplementary Table 7. Effect of using MoCo v2 on the fundus model performance for predicting any DR progression in the internal test set …………………………………………………………10

Supplementary Fig. 1. Impact of image resolution on the predictive performance of the fundus model in the internal test set.………………………………………..…………………………………..11

Supplementary Table 1. Performance of predicting DR progression in the fundus model with or without addition of HbA1c level at first follow-up visit by different glycemic control statuses in the internal test set.

Glycemic control	Fundus model		Fundus model + HbA1c at the first follow-up visit	
	AUC (95%CI)	C-index (95%CI)	AUC (95%CI)	C-index (95%CI)
Optimal	0.843 (0.781, 0.900)	0.838 (0.778, 0.893)	0.845 (0.784, 0.904)	0.840 (0.782, 0.897)
Suboptimal	0.806 (0.759, 0.848)	0.804 (0.759, 0.846)	0.813 (0.768, 0.856)	0.812 (0.768, 0.853)
Poor	0.838 (0.795, 0.878)	0.836 (0.795, 0.875)	0.841 (0.802, 0.881)	0.839 (0.801, 0.878)

Optimal glycemic control, HbA1c <7% throughout five-year follow-up;
Suboptimal glycemic control, HbA1c <7% in some visits and ≥7% in the other visits;
Poor glycemic control, HbA1c ≥7% throughout five-year follow-up.

Supplementary Table 2. Clinical characteristics of participants in the Chinese real-world study at baseline and follow-up.

	Baseline		
	Integrated Management Group (IM)	Non-integrated Management Group (Non-IM)	P value
Number of participants	538	1647	
Number of eyes	1076	3294	
Age (years)	55.62 ± 9.39	54.66 ± 9.96	0.051
Gender (Male:Female)	472:66	1361:286	0.005
Smoking (number, rate)	304, 56.51%	962, 58.41%	0.437
Drinking (number, rate)	361, 67.10%	1072, 65.09%	0.394
BMI (kg/m^2)	26.05 ± 3.20	26.48 ± 3.44	0.048
Waist (cm)	88.37 ± 9.19	89.52 ± 8.71	0.011
Hip (cm)	96.17 ± 6.14	96.53 ± 6.70	0.287
SBP (mmHg)	126.40 ± 13.19	131.19 ± 17.23	<0.001
DBP (mmHg)	74.74 ± 10.19	77.17 ± 11.28	<0.001
TC (mmol/L)	4.45 ± 0.85	5.18 ± 1.05	<0.001
TG (mmol/L)	2.07 ± 1.56	2.19 ± 1.43	0.123
HDL-C (mmol/L)	1.22 ± 0.31	1.24 ± 0.32	0.173
LDL-C (mmol/L)	2.60 ± 0.60	3.23 ± 0.82	<0.001
HbA1c (%)	6.52 ± 0.66	7.21 ± 1.35	<0.001
FPG (mmol/L)	7.19 ± 1.24	7.92 ± 2.21	<0.001
Serum insulin (pmol/L)	66.99 ± 43.98	68.06 ± 47.72	0.658
Serum C peptide (ng/mL)	2.81 ± 1.30	2.79 ± 1.00	0.877
GA (%)	15.61 ± 2.25	17.38 ± 4.15	<0.001
Scr (μmol/L)	76.46 ± 15.59	74.47 ± 17.68	0.019
BUN (mmol/L)	5.85 ± 1.39	5.86 ± 1.42	0.868
Serum uric acid (umol/L)	364.07 ± 87.14	386.77 ± 89.67	0.947
hsCRP (mg/L)	1.60 ± 2.51	1.75 ± 2.21	0.185
TnT (pg/mL)	2.26 ± 4.00	2.23 ± 4.50	0.942
Eyes with DR (number, prevalence rate)	92, 8.55%	344, 10.44%	0.072

	Follow-up		
	Integrated Management Group (IM)	Non-integrated Management Group (Non-IM)	P value
Number of participants	538	1647	
Number of eyes	1076	3294	
Age (years)	60.62 ± 9.39	59.66 ± 9.96	0.051
Gender (Male:Female)	472:66	1361:286	0.005
Smoking (number, rate)	302, 56.13%	969, 58.83%	0.270
Drinking (number, rate)	352, 65.43%	1081, 65.63%	0.930
BMI (kg/m^2)	25.97 ± 3.03	26.40 ± 3.46	0.013
Waist (cm)	88.64 ± 7.97	89.56 ± 8.97	0.042
Hip (cm)	96.24 ± 5.58	96.92 ± 6.26	0.022
SBP (mmHg)	126.93 ± 12.99	131.19 ± 16.36	<0.001
DBP (mmHg)	74.90 ± 9.77	77.36 ± 10.71	<0.001
TC (mmol/L)	4.31 ± 0.73	5.35 ± 0.99	<0.001
TG (mmol/L)	2.22 ± 1.73	2.45 ± 1.56	0.004
HDL-C (mmol/L)	1.23 ± 0.34	1.25 ± 0.33	0.083
LDL-C (mmol/L)	2.37 ± 0.49	3.33 ± 0.81	<0.001
HbA1c (%)	6.31 ± 0.48	7.18 ± 1.35	<0.001
FPG (mmol/L)	7.06 ± 1.09	8.21 ± 2.28	<0.001
Serum insulin (pmol/L)	67.66 ± 42.67	69.18 ± 45.91	0.514
Serum C peptide (ng/mL)	2.90 ± 2.07	2.82 ± 1.11	0.675
GA (%)	15.53 ± 2.13	17.67 ± 4.17	<0.001
Scr (μmol/L)	77.35 ± 15.51	75.64 ± 18.52	0.054
BUN (mmol/L)	5.87 ± 1.27	5.87 ± 1.42	0.978
Serum uric acid (μmol/L)	383.84 ± 88.21	384.98 ± 95.85	0.799
hsCRP (mg/L)	1.49 ± 1.89	1.82 ± 2.14	0.001
TnT (pg/mL)	2.03 ± 3.89	1.69 ± 3.99	0.419
Eyes with DR (number, prevalence rate)	165, 15.33%	585, 17.76%	0.067
Eyes with DR progression (number, incidence)	76, 7.06%	265, 8.04%	0.297

BMI, body mass index; SBP, systolic blood pressure; DBP, diastolic blood pressure; TC, total cholesterol; TG, triglyceride; HDL-C, high-density lipoprotein cholesterol; LDL-C, low-density lipoprotein cholesterol; HbA1c, Glycosylated hemoglobin A1c; FPG, fasting plasma glucose; GA, glycosylated albumin; Scr, Serum creatinine; BUN, Blood urea nitrogen; hsCRP, Hypersensitive C-reactive protein; TnT, Troponin T.

Supplementary Table 3. Clinical characteristics of participants in the Indian real-world study at baseline and follow-up.

	Baseline		
	Integrated Management Group (IM)	Non-integrated Management Group (Non-IM)	P value
Number of participants	76	916	
Number of eyes*	146	1798	
Age (years)	55.79 ± 10.14	55.15 ± 9.41	0.573
Gender (Male:Female)	47:29	473:443	0.231
Smoking (number, rate)	8, 10.53%	94, 10.26%	0.491
BMI (kg/m^2)	25.23 ± 3.49	25.83 ± 5.17	0.351
SBP (mmHg)	137.32 ± 20.37	138.03 ± 20.31	0.769
DBP (mmHg)	80.55 ± 11.51	82.25 ± 11.21	0.205
TG (mmol/L)	139.26 ± 19.95	188.24 ± 39.01	<0.001
HDL-C (mmol/L)	0.94 ± 0.25	1.01 ± 0.25	0.034
LDL-C (mmol/L)	1.94 ± 0.54	3.06 ± 0.94	<0.001
HbA1c (%)	5.92 ± 0.57	8.18 ± 2.12	<0.001
Eyes with DR (number, prevalence rate)	5, 3.42%	208, 11.57%	0.003

	Follow-up		
	Integrated Management Group (IM)	Non-integrated Management Group (Non-IM)	P value
Number of participants	76	916	
Number of eyes*	146	1798	
Age (years)	59.79 ± 10.14	59.15 ± 9.41	0.573
Gender (Male:Female)	47:29	473:443	0.231
Smoking (number, rate)	6, 7.89%	74, 8.08%	0.673
BMI (kg/m^2)	25.10 ± 3.66	25.44 ± 4.22	0.505
SBP (mmHg)	131.76 ± 16.26	133.47 ± 18.65	0.439
DBP (mmHg)	77.26 ± 8.71	78.19 ± 9.29	0.402
TG (mmol/L)	100.17 ± 66.17	117.26 ± 71.21	0.044
HDL-C (mmol/L)	36.89 ± 14.60	38.81 ± 11.25	0.165
LDL-C (mmol/L)	74.59 ± 18.09	104.23 ± 33.98	<0.001
HbA1c (%)	5.71 ± 0.83	7.38 ± 1.71	<0.001
Eyes with DR (number, prevalence rate)	8, 5.48%	270, 15.02%	0.001
Eyes with DR progression (number, incidence)	8, 5.48%	189, 10.51%	0.212

BMI, body mass index; SBP, systolic blood pressure; DBP, diastolic blood pressure; TG, triglyceride; HDL-C, high-density lipoprotein cholesterol; LDL-C, low-density lipoprotein cholesterol; HbA1c, Glycosylated hemoglobin A1c.

*Only eyes with gradable fundus images in both baseline and follow-up visits in the Sankara Nethralaya-Diabetic Retinopathy Epidemiology and Molecular Genetics Study were included.

Supplementary Table 4. Baseline retinal vascular measures between participants with and without any progression of DR, incidence of DR, referable DR, and VTDR.

	Any progression of DR			Incidence of DR			Incidence of referable DR			Incidence of VTDR		
	Without	With	P value	Without	With	P value	Without	With	P value	Without	With	P value
$CRAE_B$	236.283±61.25	239.891±70.69	0.449	235.405±61.03	241.207±70.61	0.263	235.504±61.39	249.576±69.75	0.035	236.546±61.82	239.299±67.34	0.783
$CRVE_B$	321.197±80.56	327.113±92.59	0.344	320.286±80.34	330.446±92.31	0.134	320.395±80.72	340.082±91.90	0.011	321.766±81.20	322.632±88.27	0.947
AVR_B	0.742±0.08	0.738±0.08	0.490	0.741±0.08	0.734±0.08	0.254	0.741±0.08	0.739±0.09	0.818	0.741±0.08	0.749±0.09	0.547
$CRAE_C$	224.195±60.15	228.507±67.50	0.345	223.388±59.95	229.697±67.03	0.201	223.493±60.18	238.174±66.82	0.011	224.520±60.58	228.384±68.29	0.693
$CRVE_C$	300.315±78.07	307.636±89.64	0.227	299.478±77.88	311.037±89.60	0.080	299.636±78.20	321.001±89.64	0.013	300.972±78.73	306.891±85.86	0.641
AVR_C	0.753±0.08	0.750±0.08	0.480	0.753±0.08	0.746±0.08	0.219	0.753±0.08	0.749±0.08	0.606	0.753±0.08	0.746±0.07	0.603
FD_C	1.240±0.04	1.243±0.04	0.364	1.240±0.04	1.243±0.04	0.303	1.240±0.04	1.250±0.04	0.006	1.240±0.04	1.238±0.05	0.726
AFD_C	1.104±0.04	1.103±0.05	0.914	1.103±0.04	1.103±0.04	0.919	1.103±0.04	1.109±0.04	0.161	1.104±0.04	1.104±0.05	0.992
VFD_C	1.095±0.04	1.101±0.04	0.023	1.095±0.04	1.102±0.04	0.014	1.095±0.04	1.109±0.04	<0.001	1.095±0.04	1.092±0.04	0.608

$CRAE_B$, central retinal artery equivalent (CRAE) in Zone B; $CRVE_B$, central retinal vein equivalent (CRVE) in Zone B; AVR_B, arteriolar-to-venular diameter ratio in Zone B; $CRAE_C$, CRAE in Zone C; $CRVE_C$, CRVE in Zone C; AVR_C, arteriolar-to-venular diameter ratio in Zone C; FD_C, total vascular fractal dimension in Zone C; AFD_C, arteriolar fractal dimension in Zone C; VFD_C, venular fractal dimension in Zone C.

Supplementary Table 5. Relationships between retinal vascular measures (per SD increase) and any progression of DR, incidence of DR, referable DR, and VTDR.

	Any progression of DR		Incidence of DR		Incidence of referable DR		Incidence of VTDR	
	HR (95% CI)	P value	HR (95% CI)	P value	HR (95% CI)	P value	HR (95% CI)	P value
$CRAE_B$	1.070 (0.939,1.219)	0.311	1.111 (0.962,1.283)	0.151	1.269 (1.047,1.537)	0.015	0.992 (0.727,1.352)	0.958
$CRVE_B$	1.098 (0.961,1.254)	0.170	1.166 (1.006,1.351)	0.041	1.318 (1.081,1.608)	0.006	0.982 (0.720,1.339)	0.909
AVR_B	0.941 (0.827,1.071)	0.359	0.901 (0.782,1.039)	0.153	0.955 (0.796,1.145)	0.617	1.034 (0.769,1.389)	0.825
$CRAE_C$	1.085 (0.952,1.237)	0.221	1.126 (0.975,1.301)	0.105	1.293 (1.067,1.567)	0.009	1.020 (0.747,1.391)	0.903
$CRVE_C$	1.117 (0.978,1.277)	0.103	1.190 (1.026,1.379)	0.021	1.357 (1.111,1.656)	0.003	1.042 (0.761,1.428)	0.797
AVR_C	0.950 (0.835,1.082)	0.442	0.905 (0.784,1.045)	0.173	0.944 (0.784,1.136)	0.542	0.896 (0.649,1.237)	0.504
FD_C	1.065 (0.937,1.210)	0.338	1.080 (0.939,1.242)	0.282	1.294 (1.077,1.555)	0.006	0.970 (0.716,1.313)	0.842
AFD_C	0.991 (0.872,1.126)	0.885	0.992 (0.863,1.140)	0.909	1.140 (0.950,1.368)	0.159	1.006 (0.742,1.363)	0.970
VFD_C	1.166 (1.026,1.326)	0.018	1.189 (1.034,1.366)	0.015	1.443 (1.204,1.730)	<0.001	0.962 (0.707,1.307)	0.802

Data are presented as HR (95% CI). HRs were estimated as per SD increase.

All groups were adjusted for age at baseline.

$CRAE_B$, central retinal artery equivalent (CRAE) in Zone B; $CRVE_B$, central retinal vein equivalent (CRVE) in Zone B; AVR_B, arteriolar-to-venular diameter ratio in Zone B; $CRAE_C$, CRAE in Zone C; $CRVE_C$, CRVE in Zone C; AVR_C, arteriolar-to-venular diameter ratio in Zone C; FD_C, total vascular fractal dimension in Zone C; AFD_C, arteriolar fractal dimension in Zone C; VFD_C, venular fractal dimension in Zone C.

Supplementary Table 6. Performance of adding retinal vascular geometry to the metadata model for predictive tasks in the internal test set.

Outcome	Metadata model	Metadata model + $CRAE_B$ + $CRVE_B$ + $CRAE_C$ + $CRVE_C$ + VFD_C	Fundus model	P value
Any progression of DR	0.699 (0.669, 0.728)	0.717 (0.686, 0.747)	0.826 (0.797, 0.853)	<0.001
Incidence of DR	0.707 (0.672, 0.738)	0.725 (0.693, 0.757)	0.828 (0.798, 0.854)	<0.001
Incidence of referable DR	0.701 (0.658, 0.742)	0.726 (0.684, 0.765)	0.822 (0.785, 0.855)	<0.001
Incidence of VTDR	0.712 (0.637, 0.779)	0.734 (0.655, 0.808)	0.825 (0.758, 0.880)	0.067

P value: Metadata model + $CRAE_B$ + $CRVE_B$ + $CRAE_C$ + $CRVE_C$ + VFD_C *vs*. Fundus model
$CRAE_B$, central retinal artery equivalent (CRAE) in Zone B; $CRVE_B$, central retinal vein equivalent (CRVE) in Zone B; $CRAE_C$, CRAE in Zone C; $CRVE_C$, CRVE in Zone C; VFD_C, venular fractal dimension in Zone C.

Supplementary Table 7. Effect of using MoCo v2 on the fundus model performance for predicting any DR progression in the internal test set.

	AUC (95%CI)	C-index (95%CI)
Fundus model without MoCo v2	0.786 (0.759, 0.812)	0.777 (0.751, 0.801)
Fundus model with MoCo v2	0.826 (0.797, 0.853)	0.823 (0.796, 0.850)

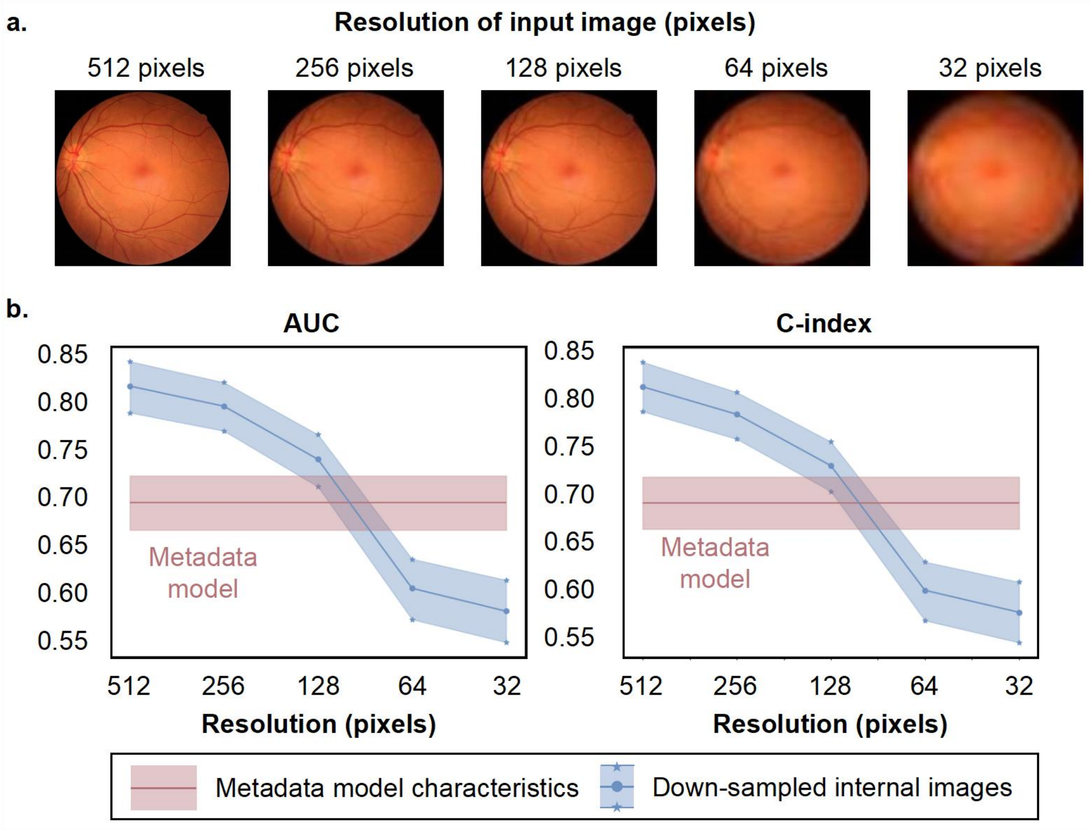

Supplementary Fig. 1. Impact of image resolution on the predictive performance of the fundus model in the internal test set. a, Sample images of different resolutions. Images were down-sampled to a certain size (for example, "32 pixels" indicates 32 × 32 pixels) and then up-sampled to the same original input size of 512 × 512 pixels. **b,** AUCs and C-indexes corresponding to different input image resolutions. Data are presented as mean values (lower bound of 95% CI, upper bound of 95% CI) computed using the DeLong method. Shaded areas in **b** are 95% CIs.
