# Supplementary material for: A deep learning system for predicting time to progression of diabetic retinopathy
Source: Nat Med. 2024 Jan 4;30(2):584–94. doi: 10.1038/s41591-023-02702-z (PMC10878973; doi:10.1038/s41591-023-02702-z)
Supplement: Supplementary file 2 — Reporting Summary [file 41591_2023_2702_MOESM2_ESM.pdf]

nature portfolio

Corresponding author(s): Bin Sheng, Huating Li, Tien Yin Wong, Weiping Jia

Last updated by author(s): Nov 6, 2023

Reporting Summary

Nature Portfolio wishes to improve the reproducibility of the work that we publish. This form provides structure for consistency and transparency in reporting. For further information on Nature Portfolio policies, see our Editorial Policies and the Editorial Policy Checklist.

Statistics

For all statistical analyses, confirm that the following items are present in the figure legend, table legend, main text, or Methods section.

n/a	Confirmed	
☐	☒	The exact sample size (*n*) for each experimental group/condition, given as a discrete number and unit of measurement
☐	☒	A statement on whether measurements were taken from distinct samples or whether the same sample was measured repeatedly
☐	☒	The statistical test(s) used AND whether they are one- or two-sided<br>*Only common tests should be described solely by name; describe more complex techniques in the Methods section.*
☒	☐	A description of all covariates tested
☒	☐	A description of any assumptions or corrections, such as tests of normality and adjustment for multiple comparisons
☐	☒	A full description of the statistical parameters including central tendency (e.g. means) or other basic estimates (e.g. regression coefficient) AND variation (e.g. standard deviation) or associated estimates of uncertainty (e.g. confidence intervals)
☐	☒	For null hypothesis testing, the test statistic (e.g. *F*, *t*, *r*) with confidence intervals, effect sizes, degrees of freedom and *P* value noted<br>*Give P values as exact values whenever suitable.*
☒	☐	For Bayesian analysis, information on the choice of priors and Markov chain Monte Carlo settings
☐	☒	For hierarchical and complex designs, identification of the appropriate level for tests and full reporting of outcomes
☒	☐	Estimates of effect sizes (e.g. Cohen's *d*, Pearson's *r*), indicating how they were calculated

Our web collection on statistics for biologists contains articles on many of the points above.

Software and code

Policy information about availability of computer code

Data collection	No special software was used for data collection.
Data analysis	Python version 3.9.0 (Python Software Foundation, Delaware, United States) was used for all statistical analyses in this study. The following third-party python packages were used: Pytorch version 2.0.1 (Facebook, Massachusetts, United States) was used for convolutional neural network computing. Scikit-learn version 1.3.0 (David Cournapeau, California, United States) was used for calculating AUC. NumPy version 1.25.2 (Travis Oliphant, Texas, United States) was used for calculating C-index, Brier score. Lifelines version 0.27.7 (Cameron Davidson-Pilon, Canada) was used for survival analysis. Singapore I vessel assessment software version 4.0 (National University of Singapore, Singapore) was used to quantify retinal vascular variables. The code being used in the current study for developing the algorithm is provided at https://github.com/drpredict/DeepDR_Plus.

For manuscripts utilizing custom algorithms or software that are central to the research but not yet described in published literature, software must be made available to editors and reviewers. We strongly encourage code deposition in a community repository (e.g. GitHub). See the Nature Portfolio guidelines for submitting code & software for further information.

Data

Policy information about availability of data

All manuscripts must include a data availability statement. This statement should provide the following information, where applicable:

- Accession codes, unique identifiers, or web links for publicly available datasets
- A description of any restrictions on data availability
- For clinical datasets or third party data, please ensure that the statement adheres to our policy

Individual-level patient data can be accessible with the consent of Data Management Committee from institutions and are not publicly available. Request for the non-profit use of the fundus images and related clinical information should be sent to Weiping Jia or Tien Yin Wong. The Data Management Committee will then review all the requests and grant (if successful). A formal data transfer agreement will be required upon approval. Generally, all these requests for access to the data will be responded to within 1month. All data shared will be de-identified. For the reproduction of our algorithm code, we have also deposited a minimum dataset at Zenodo (https://zenodo.org/records/10076339), which is publicly available for scientific research and non-commercial use.

Human research participants

Policy information about studies involving human research participants and Sex and Gender in Research.

Reporting on sex and gender	For the 19100 participants in DRPS, 15.86% were females; for the 2,141 participants in ECHM, 38.58% were females; for the 971 participants in WTHM, 32.23% were females; for the 1,194 participants in NDSP, 61.89% were females; for the 337 participants in CUHK-STDR, 50.15% were females; for the 307 participants in PUDM, 42.67% were females; for the 1,699 participants in SEED, 50.15% were females; for the 3,284 participants in SiDRP, 50.58% were females; for the 835 participants in BJHC, 47.90% were females.
Population characteristics	Patients with diabetes who are 18 years of age or older and have fundus images and clinical metadata were recruited retrospectively from multiple hospitals and community hospitals.
Recruitment	Subjects who have received fundus examination and have fundus images were recruited from multiple hospitals and community hospitals before 31 Dec 2022. The data for the model training collected from Chinese subjects, might not be representative for the generalized population, potentially introducing biases.
Ethics oversight	The study was approved by the Ethics Committee of Shanghai Sixth People's Hospital.

Note that full information on the approval of the study protocol must also be provided in the manuscript.

Field-specific reporting

Please select the one below that is the best fit for your research. If you are not sure, read the appropriate sections before making your selection.

[x] Life sciences [ ] Behavioural & social sciences [ ] Ecological, evolutionary & environmental sciences

For a reference copy of the document with all sections, see nature.com/documents/nr-reporting-summary-flat.pdf

Life sciences study design

All studies must disclose on these points even when the disclosure is negative.

Sample size	We developed DeepDR Plus system for predicting DR progression using a total of 76,400 retinal fundus images from 19,100 diabetic patients and we validated the system by 42,558 retinal fundus images from 10,768 diabetic patients. The sample size was determined by the data availability.
Data exclusions	Retinal images of poor image quality were excluded.
Replication	Replication was not relevant. We used eight independent validation cohorts to test the models, and the models achieved similar performances in the external validation sets.
Randomization	Samples were randomly allocated to the developing and validation datasets.
Blinding	During the data processing, all data was first de-identified to remove any patient related information.

Reporting for specific materials, systems and methods

We require information from authors about some types of materials, experimental systems and methods used in many studies. Here, indicate whether each material, system or method listed is relevant to your study. If you are not sure if a list item applies to your research, read the appropriate section before selecting a response.

Materials & experimental systems

n/a	Involved in the study
☒	☐ Antibodies
☒	☐ Eukaryotic cell lines
☒	☐ Palaeontology and archaeology
☒	☐ Animals and other organisms
☐	☒ Clinical data
☒	☐ Dual use research of concern

Methods

n/a	Involved in the study
☒	☐ ChIP-seq
☒	☐ Flow cytometry
☒	☐ MRI-based neuroimaging

Clinical data

Policy information about clinical studies

All manuscripts should comply with the ICMJE guidelines for publication of clinical research and a completed CONSORT checklist must be included with all submissions.

Clinical trial registration	The study was registered on Chinese Clinical Trial Registry (http://www.chictr.org.cn/). Registration number: ChiCTR2300069400.
Study protocol	Study protocols can be found in http://www.chictr.org.cn/.
Data collection	Fundus images in the DRPS cohort were collected in Wuxi and Shanghai between 2015 and 2022. Fundus images in the SIM cohort were collected in Shanghai between 2014 and 2017. Fundus images in the ECHM cohort were collected in Wuxi between 2006 and 2016. Fundus images in the WTHM cohort were collected in Wuhan between 2010 and 2021. Fundus images in the NDSP were collected in Nicheng Community in 2013 and 2018.Fundus images in the CUHK-STDR were collected in HongKong between 2015 and 2021. Fundus images in PUDM were collected in Beijing between 2010 and 2016. Fundus images in SEED cohort were collected in Singapore between 2004 and 2017. Fundus images in SiDRP cohort were collected in Singapore between 2010 and 2015. Fundus images in the BJHC were collected in Beijing between 2014 and 2020.
Outcomes	The primary outcome was any DR progression. The secondary outcome was the progression from no retinopathy to DR, non-referable DR to referable DR, and non-vision-threatening DR to vision-threatening DR. The diagnosis and classification of DR were evaluated by ophthalmologists according to the ICDRDSS22.
